# Luciferase-Specific Coelenterazine Analogues for Optical Contamination-Free Bioassays

**DOI:** 10.1038/s41598-017-00955-6

**Published:** 2017-04-19

**Authors:** Ryo Nishihara, Masahiro Abe, Shigeru Nishiyama, Daniel Citterio, Koji Suzuki, Sung Bae Kim

**Affiliations:** 1 Department of Applied Chemistry, Faculty of Science and Technology, Keio University, 3-14-1 Hiyoshi, Kohoku-ku, Yokohama Kanagawa, 223-8522 Japan; 2 Research Institute for Environmental Management Technology, National Institute of Advanced Industrial Science and Technology (AIST), 16-1 Onogawa, Tsukuba, 305-8569 Japan

## Abstract

Spectral overlaps among the multiple optical readouts commonly cause optical contamination in fluorescence and bioluminescence. To tackle this issue, we created five-different lineages of coelenterazine (CTZ) analogues designed to selectively illuminate a specific luciferase with unique luciferase selectivity. In the attempt, we found that CTZ analogues with ethynyl or styryl groups display dramatically biased bioluminescence to specific luciferases and pHs by modifying the functional groups at the C-2 and C-6 positions of the imidazopyradinone backbone of CTZ. The optical contamination-free feature was exemplified with the luciferase-specific CTZ analogues, which illuminated anti-estrogenic and rapamycin activities in a mixture of optical probes. This unique bioluminescence platform has great potential for specific and high throughput imaging of multiple optical readouts in bioassays without optical contamination.

## Introduction

Luciferases are a family of light-generating proteins found in a large variety of insects, marine organisms and prokaryotes^1^. Beetle luciferases, including firefly luciferase (FLuc), mediate oxidative decarboxylation of D-luciferin in the presence of ATP, Mg^2+^, and O_2_; whereas many luciferases from marine organisms utilize coelenterazine (CTZ), and require only O_2_ for catalysis^2^. Luciferases have peak emissions ranging from blue to red, ca. 400–620 nm^2^. However, the enzymes’ emission bandwidths are broad and significantly overlap in the yellow-green region^3^, which creates spectral overlap when multiple marine luciferases are incorporated into the same reaction condition and concurrently activated by a pan-specific coelenterazine substrate.

Overlapping emissions from luciferases impair multiplex imaging of *in vivo* reporter assays, resulting in optical signal contamination. In contrast, fluorescent proteins have a broader color palette, but suffer from strong background due to autofluorescence. Tactical algorithms for unmixing overlapping multiple optical spectra, by a process of spectral unmixing calculations and deconvolution, have been developed^4, 5^; however, they have not provided a fundamental solution to this problem. In addition, near-infrared fluorescent proteins (iRFPs)^6^, several variants of beetle and marine luciferases^7, 8^, and red- and blue-shifted analogues of CTZ^3, 9^ have been studied, but so far have been ineffective in eliminating signal contamination in multiplex assays due to insufficient spectral shift, and/or low intensity light emission.

**Figure 1:**
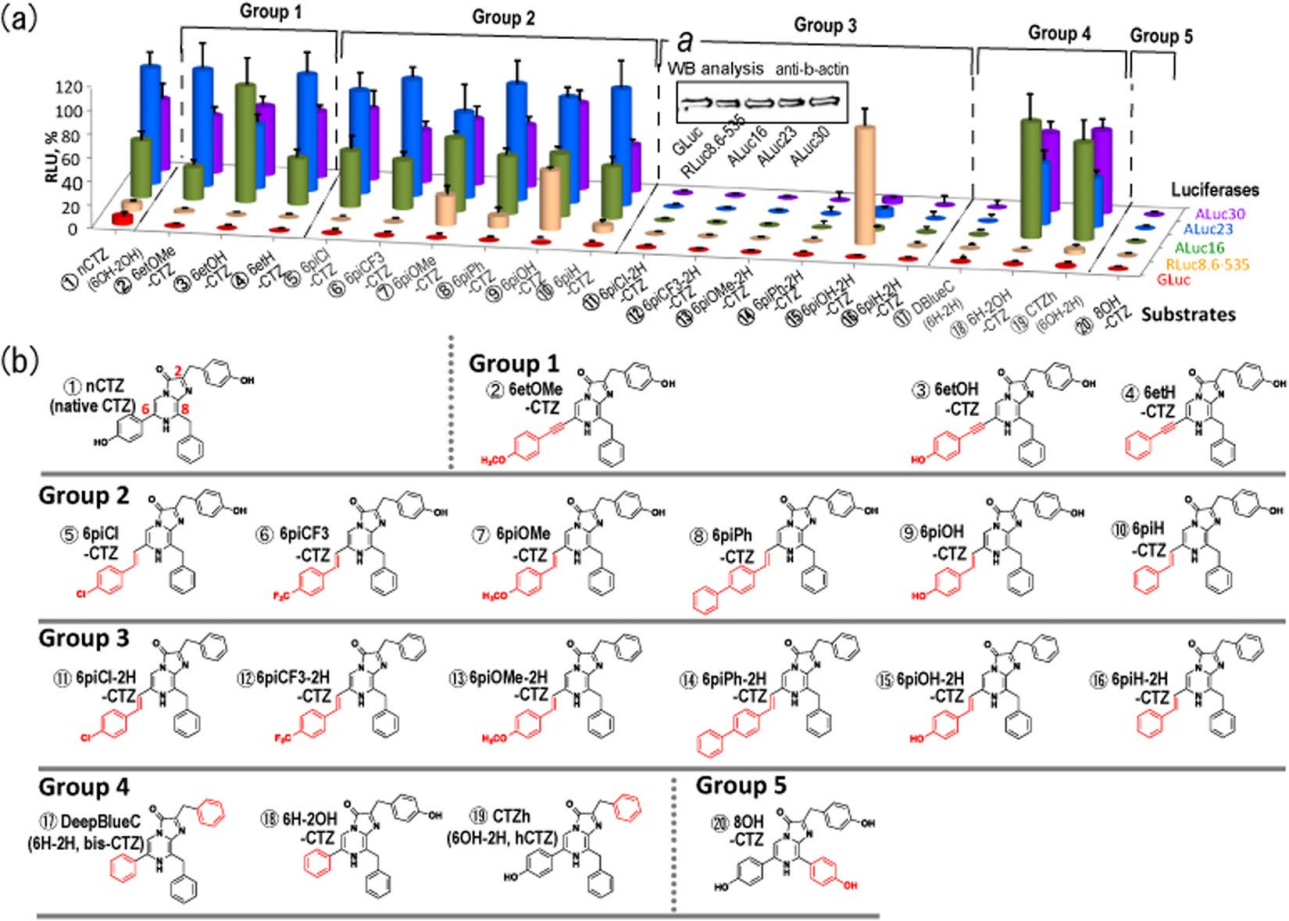
(**a**) Relative optical intensities of marine luciferases to the synthetic luciferins. The optical intensities of five marine luciferases to each luciferin were normalized to the maximal intensity. The synthetic luciferins were grouped into five categories according to their chemical structures. Inset *a* shows a Western blot analysis indicating relative protein amounts between the marine luciferases. The house-keeping protein, β-actin, was stained with a specific antibody. **(b**) The detailed chemical structures of synthetic coelenterazine analogues. Compound ① (native CTZ; nCTZ) and its derivatives are categorized into five groups: Group 1 contains an ethynyl group at the C-6 position. The distinctive feature between groups 2 and 3 is the presence or absence of the hydroxyl (OH) group at the C-2 phenyl ring. Both groups 2 and 3 share extension of the double bond conjugated system at the C-6 position. Group 4 analogues lack the OH group at the C-2 and/or C-6 positions, upon comparison with nCTZ. Group 5 has an OH group at the C8 position. The characteristic functional groups in the chemical structures are highlighted in red.

In order to take advantage of the high sensitivity and low background of marine luciferases, our objective was to find a solution to spectral overlap by synthesizing new CTZ analogues with isotype specificity. Here we studied 20 CTZ analogues, including 15 newly synthesized analogues, with modifications to the C-2 and C-6 functional groups (Fig. 1; Suppl. Fig. 1) according to the procedure in Suppl. Figs 3 and 4, which enabled us to develop luciferase-specific analogues. In addition, we integrated this substrate-specific activation of luciferases into a two-switch assay system that further increased specificity by utilizing two single-chain probes, previously reported by us^10, 11^. The application of this system enabled us to selectively activate a specific luciferase, thereby preventing spectral overlap that is usually caused by the concurrent activation of more than one luciferase. The system was designed to carry *Renilla reniformis* luciferase 8 (RLuc8) and artificial luciferases (ALucs), where ALucs were previously fabricated by 3 steps: (i) formation of an artificial sequence by extracting frequently occurring amino acid sequences from the aligned sequences of copepod luciferases, (ii) single-sequence alignment (SSA) of the artificial sequence for highlighting the repeated catalytic domains, and (iii) increase of the sequential homology between the repeated catalytic domains. The brief process was illustrated in Suppl. Fig. 9 ^12^. The maximal sequential identities of ALuc16 and ALuc25 for instance are 81% and 72% of that of *Metridia pacifica* luciferase, according to NCBI Blast Alignment Search, ver. BLASTP 2.2.27+. Their crystallographic structures are not determined yet. This method provides a breakthrough for overcoming spectral overlap by specifically determining multiple optical readouts in mixture with high throughput, and facilitates contamination-free bioassays and molecular imaging.

## Results

### Structural analyses of the marine luciferase–luciferin interactions

In order to help elucidate the mechanisms of substrate specificity for *Renilla reniformis* luciferase (RLuc) and artificial luciferases (ALucs), and considering the absence of any crystal structures of ALucs, we initially inspected the interactions between RLuc8 and coelenteramide in the published crystal structure, PDB ID: 2PSJ^3^. The bound coelenteramide (oxidized product of CTZ) differs in structure to native CTZ (Suppl. Fig. 2); however, the functional groups at the C-2 and C-6 positions of the imidazopyrazine backbone are unchanged. The C-6 hydroxyphenyl inserts into the active site pocket, and the *para*-hydroxyl (the hydroxy group at the 4 position of the phenyl ring) interacts with His285 and Glu144 at the base of the pocket, which are reported to act as a catalytic triad in conjunction with Asp120^13^. Residues Trp156, Val185, Lys189, Phe261, and Phe262 form a hydrophobic annulus around the C-6 phenyl group. By increasing the steric bulk at the C-6 position, we predicted that steric hindrance would inhibit binding to RLuc, and that, due to the low sequence identity between RLuc and ALucs, ca. 17%, ALucs might be able to accommodate a bulkier moiety if they contain variant residues in their active site pockets. In contrast, the C-2 hydroxyphenyl group of the bound coelenteramide protrudes from the active site pocket, and forms hydrophobic interactions with Leu165, and Phe180.

### Determination of luciferase specificity of newly synthesized CTZ analogues

Five-different lineages of CTZ analogues were synthesized to make a new breakthrough for specific imaging of intracellular molecular events in mammalian cells (Fig. 1).

To date, most research has focused on the synthesis of C-2, C-6 and C-8 substituents of CTZ in combination with analyses utilizing marine luciferases^14, 15^. However, most of these reported CTZ analogues failed to induce bright bioluminescence with RLuc, whereas we successfully reported that C-6 position-modified CTZ analogues exerted significantly increased bioluminescence with RLuc8 and RLuc8.6-535 ^9^. On the basis of the structural analysis above, we attempted to modify both C-2 and C-6 positions in the hope of rationally altering the luciferase selectivity.

Among synthesized CTZ analogues, high ALuc specificity was found with Group 1 (i.e., 6et-X-CTZ analogues, where “6et” means an ethynyl group at the C-6 position and “X” symbols any functional group), which is characteristic in the ethynyl group at the C-6 position, compared to the other analogues (Fig. 1 and Suppl. Fig. 1(a)). In contrast, the same CTZ analogues of Group 1 did not produce notable bioluminescence with the other conventional marine luciferases, GLuc and RLuc8.6-535: e.g., ALuc16 and ALuc23 exerted 1530-fold (±185) and 3378-fold (±382) stronger bioluminescence intensity than GLuc with 6etOMe-CTZ. Similarly, RLuc8.6-535 did not show any considerable optical intensity with the CTZ analogues in Group 1.

6**et**OMe-CTZ and 6**et**OH-CTZ in Group 1 show high selectivity only to ALucs, whereas 6**pi**OMe-CTZ and 6**pi**OH-CTZ in Group 2 are accommodated into both RLuc8.6-535 and ALucs. It is considered that the double bond extension and orientation at the C-6 position determines the structural preference for both RLuc8.6-535 and ALucs.

CTZ analogues of Group 2 are characterized by a styryl group at the C-6 position and an OH group at the C-2 position (i.e., 6pi-X-CTZ, where “6pi” means a styryl group and “X” indicates any functional group). In this group, 6pi**Cl**-CTZ and 6pi**CF**_3_-CTZ show high selectivity to ALuc only. 6pi**OH**-CTZ of Group 2 interestingly exhibited biased selectivity to both ALucs and RLuc8.6-535, but not to GLuc. The structural comparison between 6**pi** **OH**-CTZ of Group 2 and 6**H**-2OH-CTZ of Group 4 reveals that RLuc8.6-535 is strongly influenced by not only the presence or absence of the OH group at the C-6 position, but also the length of the functional group at the C-6 position. In contrast to RLuc8.6-535, ALucs can easily accommodate bulky and extended chemical structures of the sidechain at the C-6 position, which is considered to be located at the loose moiety of ALucs.

Group 3 is unique in the point that all derivatives share a styryl group at the C-6 position of the imidazopyradinone backbone and lack the hydroxyl (OH) group at the C-2 position (the phenol group). Group 3 may be abbreviated 6pi-X-2H-CTZ, where “6pi” means a styryl group at the C-6 position and “X” indicates any functional group. Furthermore, “2H” shows a benzyl group at the C-2 position. All the CTZ analogues of this group, except 6piOH-2H-CTZ, failed to develop bioluminescence with all the examined marine luciferases. 6piOH-2H-CTZ of Group 3 interestingly luminesced selectively with RLuc8.6-535. 6piOH-2H-CTZ with RLuc8.6-535 efficiently emitted 1014-fold (±149) and 38-fold (±5) stronger bioluminescence intensity than with GLuc and ALuc16, respectively.

The comparison of the chemical structure of 6piOH-**2H**-CTZ with that of 6piOH-CTZ reveals that the OH group at the C-2 position is a key functional group in binding to ALucs, but is not essential for interaction with RLuc8.6-535. It is interpreted in a way that the C-2 position is located at the loose moiety of RLuc8.6-535 and thus less influential, different from the case of ALuc (Suppl. Fig. 2).

**Figure 2:**
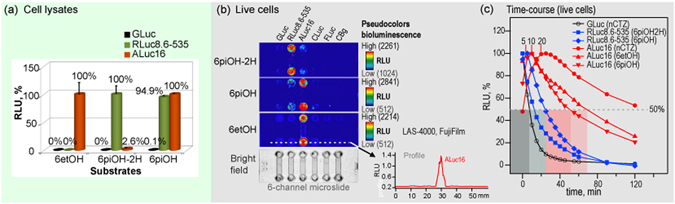
Biased selectivity of the synthetic luciferins to marine luciferases. (**a**) The relative selectivity of representative synthetic luciferins according to luciferases in cell lysates. (**b**) The corresponding live-cell images on a 6-channel microslide, demonstrating the relative luciferase-selectivity of the selected coelenterazine (CTZ) analogues in living mammalian cells. The optical intensity ranges of 6piOH-2H-CTZ, 6etOH-CTZ, and 6piOH-CTZ were 1024–2261, 512–2214, 512–2841 RLU, respectively. (**c**) The time course of the optical intensities after substrate injection to the microslide channels growing live cells. The bioluminescence exhibited clearly distinctive time courses according to the chemical structures of the synthetic luciferins and the kinds of marine luciferases. The blue- and red-marked lines indicate the intensity profiles of RLuc8.6-535 and ALuc16 by time, respectively. Greatly enhancing features in the optical intensity by time were observed with ALuc16. Abbreviations: GLuc, *Gaussia ﻿princeps* luciferase; RLuc8.6-535, *Renilla reniformis* luciferase 8.6-535; ALuc16, artificial luciferase 16; CLuc, Cypridina luciferase; FLuc, firefly luciferase; CBgreen, click beetle luciferase green.

A comparison of the chemical structures between 6pi**OH**-2H-CTZ and 6pi**H**-2H-CTZ of Group 3 shows that the OH group at the C-6 position of 6piOH-2H-CTZ is essential for interacting with the residues in the active site of RLuc8.6-535. Furthermore, upon review of the chemical structures between 6piOH-**2H**-CTZ and 6piOH-CTZ, it shows that the OH group at the C-2 position is a key functional group only for ALucs, but not for RLuc8.6-535. The greatly biased optical intensities were highlighted in Fig. 2 (relative) and Table 1 (absolute).

**Table 1:** The absolute optical intensities of the selected CTZ analogues according to luciferases (n = 3).

RLU/sec/mm^2^			
	③ 6etOH-CTZ	⑮ 6piOH-2H-CTZ	⑨ 6piOH-CTZ
GLuc	**2** (±2)	**4** (±3)	**8** (±1)
RLuc8.6-535	**1** (±1)	**1014** (±149)	**6340** (±215)
ALuc16	**510** (±116)	**27** (±6)	**6680** (±148)

The optical intensities (RLU) as primary data were divided by integration time (sec) and area (mm^2^). Therefore, the unit is RLU/sec/mm^2^. The standard deviation (±s.d.) was specified in parenthesis.

CTZ derivatives of Group 4 have no OH group at the benzene rings of C-2 and/or C-6. DeepBlueC (6H-2H-CTZ in our nomenclature; or bis-CTZ in common name) developed no considerable bioluminescence with all the tested luciferases. 6H-2OH-CTZ and CTZ*h* (6OH-2H-CTZ in our nomenclature; or hCTZ in common name) showed ALuc-biased optical intensities.

The 8OH-CTZ of Group 5 showed no optical intensities with the applied luciferases.

### Evaluation of the spectral overlaps among marine and beetle luciferases

The spectral overlaps among marine and beetle luciferases was briefly demonstrated (Suppl. Fig. 1(b) inset *a*). The spectral peaks were found in the range from 480 to 560 nm. The bioluminescence spectra have broad full width at half maximum (FWHM) and thus the major portion of the spectra overlapped each other. The spectrum of RLuc8.6-535 overlaps ca. 75% and 82% with that of GLuc and ALuc16, respectively, if we suppose the optical intensities are equivalent each other. Further, the spectrum of RLuc8.6-535 is superimposed with ca. 96% and 89% on click beetle luciferase green (CBgreen) and FLuc, respectively, upon normalization of the optical intensities into percentages (%).

### Multiplex assay system for specific illumination of bioactivities of ligands

**Figure 3:**
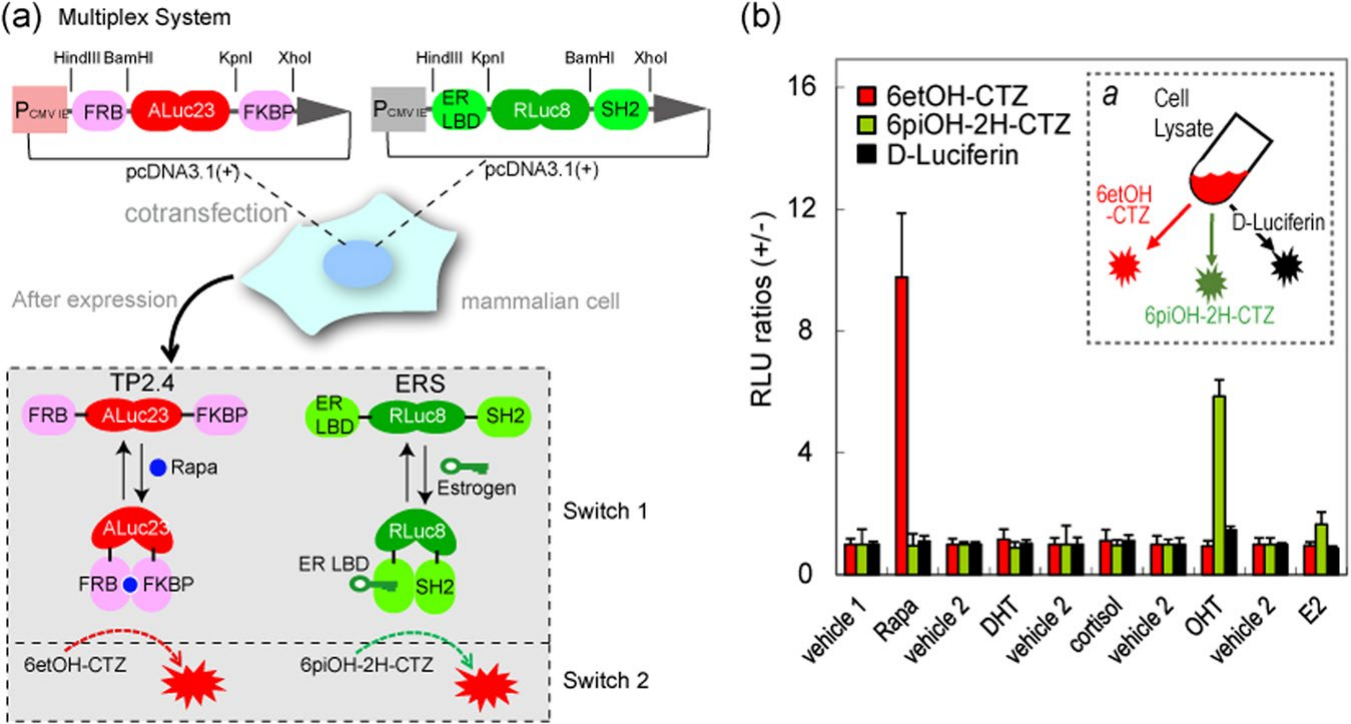
(**a**) Schematic diagram of a multiplex assay system with two pcDNA3.1(+) vectors encoding molecular tension probe 2.4 (TP2.4) and ER LBD-RLuc8-SH2 (ERS), respectively. After expression, TP2.4 and ERS coexist in mammalian cells, which are ready to illuminate ligand activity. The working mechanisms of TP2.4 and ERS are briefly illustrated in the box-highlighted Switches 1 and 2. Upon stimulation by a ligand, TP2.4 or ERS exerts an intramolecular protein-to-protein interaction (PPI) (Switch 1). This PPI enhances the sandwiched luciferase’s activity. The enhanced activities are pinpoint visualized with specific coelenterazine (CTZ) analogues (Switch 2). (**b**) Specific optical intensities of the substrates in the multiplex assay system carrying two luciferase-based probes. Conventional assays are unable to discriminate the two optical signals. However, the present multiplex system pinpoint-illuminated the rapamycin or estrogen antagonist (OHT) activities without any signal contamination. Vehicles 1 and 2 are negative controls with 0.1% alcohol and 0.1% DMSO, respectively. Inset *a* briefly illustrates the experimental procedure with the specific substrates. The live cells were firstly stimulated with rapamycin and estrogen, lysed, and pinpoint-illuminated with a specific luciferin. Abbreviations: FRB, the rapamycin-binding domain of mTOR; FKBP, FK506-binding protein; ER LBD, the ligand binding domain of human estrogen receptor; SH2, the SH2 domain of *v*-Src; Rapa, rapamycin; DHT, 5α-dihydrotestosterone; OHT, 4-hydroxytamoxifen; E2, 17β-estradiol.

A multiplex assay system was fabricated for demonstrating whether the substrates specifically illuminate bioactivities of a ligand (Fig. 3).

The optical intensities from COS-7 cells co﻿expressing ERS and TP2.4 were developed with D-Luciferin, 6etOH-CTZ, or 6piOH-2H-CTZ. Among the substrates, 6etOH-CTZ selectively elevated the luminescence intensity 9-fold, compared to that of the negative control (0.1% ethyl alcohol) only with the cell lysates stimulated by 10^−5^ M rapamycin, whereas 6piOH-2H-CTZ pinpoint-illuminated 6-fold stronger bioluminescence only with the cell lysates stimulated by 10^−5^ M OHT.

In contrast, D-luciferin did not exhibit any elevation of the optical intensities from the cell lysates. The above results demonstrate that (i) the newly synthesized substrates, 6etOH-CTZ and 6piOH-2H-CTZ, allow to pinpoint-illuminate rapamycin and ER antagonist (OHT) activities, respectively, and (ii) the ligand specificity is derived from the unique selectivity of 6etOH-CTZ and 6piOH-2H-CTZ to ALuc23 (TP2.4), and to RLuc8 (ERS), respectively.

### Prolonged bioluminescence stability of CTZ analogues in living mammalian cells

The time-course of bioluminescence intensities of the key CTZ analogues was determined with live COS-7 cells expressing GLuc, RLuc8.6-535, or ALuc16 (Fig. 3(b), Suppl. Fig. 5).

The excellent luciferase specificity of the synthesized CTZ analogues, 6etOH-CTZ and 6piOH-2H-CTZ was observed even in living mammalian cells, grown in a 6-channel microslide (Fig. 3(b)). The luciferase-specific features in live cells almost corresponded with those of the cell lysates shown in Fig. 1. Meanwhile, discordance in the results between the lysates and the live cells was also observed with 6piOH-CTZ: i.e., the 6piOH-CTZ–RLuc8.6-535 combination emitted ca. 95% of the optical intensity of the 6piOH-CTZ–ALuc16 combination in the lysate, but the same combination in the lysate showed only 20% emission of the 6piOH-CTZ–ALuc16 mixture in living mammalian cells. This difference in emission in the lysates and in the live cells, along with the pH environment, is discussed in the Discussion and Supplementary sections.

The bioluminescence emission half-lives of RLuc8.6-535 with 6piOH-2H-CTZ and 6piOH-CTZ in living COS-7 cells are found to be ca. 12.6 and 24.2 minutes, respectively. On the other hand, 6piOH-CTZ and 6etOH-CTZ showed greatly prolonged half-lives with ALuc16, which took 50.7 and 69.5 minutes to drop to half-maximal intensity during decay (Suppl. Fig. 5).

GLuc in living COS-7 cells quickly decreased its optical intensity by the addition of nCTZ and reached half-maximal optical intensity within 7.4 minutes, whereas ALuc16 showed a greatly prolonged and growing bioluminescence over time. The ALuc16–nCTZ combination reached the maximal optical intensity at ca. 20 minutes and then gradually decayed to the half-maximal intensity in ca. 140 minutes, which is approximately 18-fold longer than the GLuc–nCTZ combination, and ca. 10.5-fold more sustained than the RLuc8.6-535–6piOH-2H-CTZ combination. A growing optical profile over time was also found with ALuc16–6etOH-CTZ and ALuc16–6piOH-CTZ pairs, whose intensities reached the maximal values in 5 and 10 minutes, respectively. The live-cells were exposed to the newly synthesized CTZs for maximally 20 minutes. During the experiments, no cytotoxicity caused by the newly synthesized CTZs was observed with naked eyes.

The above results are summarized by the following: (i) the key CTZ analogues with ethynyl and styryl groups exert excellent luciferase specificity in both lysates and living mammalian cells, (ii) the key CTZ analogues with ethynyl or styryl groups allow greatly prolonged and growing profile of bioluminescence with RLuc8.6-535 and ALuc16 in living mammalian cells, and (iii) the growing profile of bioluminescence reflects the relative plasma membrane permeability of the CTZ analogues in living mammalian cells, considering that the intracellular localization of ALuc16 and RLuc8.6-535 differs in the cells and the growing profile is not observed in the cell lysates.

## Discussion

Multiplex imaging of intracellular molecular events is expected to show a key breakthrough for bioassays and molecular imaging, considering the potential efficiency and high sample throughput. However, most emission spectra of conventional reporter luciferases are superimposed on each other in the green and yellow-green regions, resulting in spectral overlaps (Suppl. Fig. 1(b) inset *a*). Spectral unmixing algorisms, optical filters, and quenching reagents have not provided a fundamental solution to overcome the spectral crosstalk and signal leakage. To tackle this issue, we examined 20 kinds of CTZ analogues (15 of which were newly synthesized), designed to selectively access the active site cavity of a luciferase, and pinpoint-illuminate bioluminescence even in a multiplex assay system. The synthesized CTZ analogues were divided into five groups according to their chemical structures, and discussed in the context of the quantitative chemical structure–bioluminescence intensity relationship (QSIR). The unique design of CTZ analogues was inspired by our review of the luciferin-binding chemistry between RLuc8 and coelenteramide in the crystal structure showing the contribution of the C-2 and C-6 positions to the luciferase selectivity: e.g., by increasing the steric bulk at the C-6 position of CTZ, the steric hindrance would inhibit the binding to RLuc8.

The experimental results with 5 lineages of CTZ analogues revealed that 6etOH-CTZ and 6piOH-2H-CTZ pinpoint-illuminated the activities of ALuc16 and RLuc8.6-535, respectively, while 6piOH-CTZ luminesced with both ALuc16 and RLuc8.6-535. The overall results allow us to conclude that the OH groups and the length at the C-2 and C-6 positions of CTZ analogues are critical determinants for the selectivity to ALuc and RLuc, respectively.

The dominant role of the C-6 position of CTZ to RLuc8 activity was previously reported^13^. The study revealed that the OH group at the C-6 position forms conserved triad binding with the residues of RLuc8, and has a key role in the activity. We independently showed that CTZ*i* carrying iodine at the C-2 position is highly selective to ALuc30. We hypothesized it is because the iodine is accommodated into a room in the active site pocket of ALuc30^16^. The above views support that the present luciferase-luciferin reactions adhere a typical host–guest chemistry.

The time course of bioluminescence in Fig. 2(c) may be discussed with the relative membrane permeability of CTZ analogues in living mammalian cells. Different from other marine luciferases, ALuc16 showed a gradually increasing bioluminescence after substrate injection with 6piOH-CTZ, 6etOH-CTZ, and nCTZ, which were found to reach the optical maxima at ca. 5, 10, and 20 minutes, respectively. In living mammalian cells, ALuc16 is sequestered into the endoplasmic reticulum (ER), which the substrates have to gain access to (meaning crossing two plasma membrane barriers). The results are interpreted as (i) the substrates reach delayed equilibrium with ALuc16 in the ER, (ii) the order of 6piOH-CTZ, 6etOH-CTZ, and nCTZ should reflect the relative plasma membrane permeability of the substrates in living mammalian cells, and (iii) ALuc16 sequestered in the ER generally reaches slower equilibrium than RLuc8.6-535 localizing in the cytosol.

RLuc8.6-535 reaches more delayed equilibrium with 6piOH-CTZ than with 6piOH-2H-CTZ, whose only structural difference is the number of the hydrophilic OH groups (Fig. 1(b)). 6piOH-CTZ comprising two OH groups should permeate the lipophilic plasma membrane with a delayed time course, compared to 6piOH-2H-CTZ carrying a single OH group.

Discrepancy in the relative optical intensities of 6piOH-CTZ in the lysates (Fig. 2(a)) and the live cells (Fig. 2(b)) may be explained by the distinctive pH environments in the lysates and the cytosol of the living cells: i.e., the pHs of the lysis buffer (Promega) and the cytosol of living mammalian cells are ca. 5.6 and neutral (pH 7.0–7.4)^17^, respectively. As shown in Suppl. Fig. 6, the optical intensity gaps between ALucs and RLuc8.6-535 are larger in the neutral range than the acidic region. Thus, it is natural that the relative intensity gap between ALuc16 and RLuc8.6-535 in living mammalian cells is larger than in the light emission-suppressive acidic condition (pH 5.6) in the lysates.

There is great merit to be had in specific and high throughput determination of multiple optical readouts in bioassays and molecular imaging. Figure 3 showed that the luciferase specificity of synthetic CTZ analogues allows the simultaneous determination of two distinct ligand activities in a mixture of single-chain probes without optical signal crosstalk. This means that two distinctive “on-off” switches work in the present multiplex assay system: i.e., the first switch is the ligand binding of the probe set, ERS and TP2.4, and the second switch is the substrate binding of the probe set (Fig. 3(a)). The multiplex system is designed to luminesce only in the case that the two switches are “on”: i.e., the probe in the system luminesces only in the coexistence of both the agonist and the specific substrate.

Taken together, the newly synthesized CTZ analogues presented here show that selective activation of luciferases can be achieved, and that this selectivity can be exploited to pinpoint-illuminate specific luciferase activity among multiple optical readouts. In addition, we tailored the specific activation of luciferases by utilizing two distinct molecular probe proteins, only able to luminesce in the combined presence of a specific CTZ analogue and a specific bioactive small molecule in a two-switch system. Although our system was unable to change the emission spectra of the luciferases, its application prevented spectral overlap, enabling high throughput screening of signal contamination-free bioassays and molecular imaging. Co-crystal structures of ALucs and RLuc8 with bound CTZ analogues would facilitate the development of even more specific analogues, and further optimization of our system. In addition, elucidation of the binding interactions between substrates and residues in the active site pockets of luciferases, could potentially be exploited by protein engineering to change the wavelength of light emission, and provide improved applications amenable to bioassays, *in vivo* imaging, and analyses of live animal models with more efficient penetration of light though tissues.

## Methods

### Design and organic synthesis of luciferase-specific CTZ analogues

RLuc and *Oplophorus* Luciferase (OLase) are reported to exert broad substrate specificities with the C-2 position-modified CTZ analogues^14^. Separately, we showed that RLuc8 and RLuc8.6-535 emit significant bioluminescence with C-6 position-modified CTZ analogues^9^. This precedent study inspired us to synthesize unique CTZ analogues modified at the C-2 and/or C-6 positions.

In this report, in order to fabricate CTZ analogues allowing a unique optical specificity to a marine luciferase, we synthesized a series of C2- and/or C-6-substituted CTZ analogues (Fig. 1). The synthetic process is described in Suppl. Figs 3 and 4, in detail. The new CTZ derivatives besides conventional CTZ analogues are categorized into five groups according to the chemical structure, for convenience (Fig. 1 and Suppl. Fig. 1). The CTZ analogues are designated hereafter with the abbreviated names.

The chemical structural characteristics of the groups are as follows: compounds of Group 1 share a common ethynyl group at the C-6 position, and a phenol group at the C-2 position. Compounds of Group 2 commonly carry a styryl group at the C-6 position, and a benzyl group at the C-2 position. Compounds of Group 3 comprise a common styryl group at the C-6 position, and a phenol group at the C-2 position. In the case of compounds of Groups 4 and 5, their funtional groups in the chemical structures are very close to the nCTZ. The only difference of Group 4 from nCTZ is whether the OH groups at the C-6 and/or C-2 positions exist or not. The structure of Group 5 is different from nCTZ in the point that the C-8 position has a phenol group.

The newly synthesized CTZ analogues are stable when stored at −30 °C, in darkness and nitrogen atmosphere. The CTZ analogues are unstable with dimethyl sulfoxide (DMSO) (Suppl. Fig. 8).

### Luciferase specificity of newly synthesized CTZ analogues

The optical specificity of the 20 CTZ analogues were determined with various marine luciferases (Fig. 1).

African green monkey kidney fibroblast-derived COS-7 cells were grown in a 96-well optical bottom plate (Thermo Scientific). The cells were transiently transfected with pcDNA 3.1(+) vector encoding GLuc, RLuc8.6-535, or a series of ALucs, and incubated in a CO_2_ incubator (Sanyo), where GLuc and ALucs are designed to commonly carry an endoplasmic reticulum (ER)-retention signal, KDEL, for their retention in the intracellular compartments after expression as explained previously^12, 16^. Sixteen hours after transfection, the cells were lysed with a lysis buffer (Promega) for 20 minutes according to the manufacturer’s instructions. Aliquots of the lysates (10 μL), on a fresh 96-well plate, were mixed with an assay solution dissolving 50 μL of a CTZ analogue (10^−4^ M, final concentration) and immediately inserted into a dark chamber (LAS-4000 (FujiFilm)) equipped with a cooled charge coupled device (CCD) camera system. The optical intensities were determined with an image acquisition software (Image Reader v2.0) and analyzed with a specific image analysis software (Multi Gauge v3.1).

The applied protein amounts of the luciferases were gauged via a Western blotting analysis (Fig. 1(a) inset *a*). COS-7 cells transiently transfected with pcDNA3.1(+) vectors encoding luciferases were washed once with PBS and lysed with an aliquot of a sample buffer carrying 10% 2-mercaptoethanol (Wako). An aliquot of each sample was electrophoresed in a pre-casted acrylamide gel (Bio-Rad), transferred to a nitrocellulose membrane (Millipore), and incubated with a mouse anti-β-actin antibody (Sigma) (primary antibodies). The membrane blots were further treated with horseradish peroxidase (HRP)-conjugated secondary antibody (GE Healthcare) and finally visualized with a chemiluminescence substrate kit (Wako).

The bioluminescence spectra of various luciferases were determined in order to measure the superimposed area (Suppl. Fig. 1(b) inset *a*). The COS-7 cells were transfected with pcDNA 3.1(+) encoding GLuc, RLuc8, RLuc8.6-535, CBgreen, FLuc, ALuc16, and ALuc23. The cells were incubated for 16 h and lysed with a lysis buffer (Promega) for marine luciferases (GLuc, RLuc8, RLuc8.6-535, ALuc16, and ALuc23), or with a Bright-Glo assay solution (Promega) containing D-luciferin for beetle luciferases (CBgreen and FLuc). An aliquot of the lysates in a microtube (10 μL) was mixed with an assay buffer carrying 10^−4^ M of native CTZ for marine luciferases, whereas all the lysates prepared for beetle luciferases were transferred to a microtube without any treatment. The microtubes were immediately transferred to the dark chamber of a precision spectrophotometer (AB-1850, ATTO), which simultaneously acquires the full visible and near infrared ranges of emitted photons (i.e., 391–789 nm). The corresponding optical spectra were determined in a 20-second integration mode. The spectra were normalized in percentages for peak emission.

### Luciferase-selective property of CTZ analogues in lysates and living mammalian cells

Luciferase specificity and kinetics of the key CTZ analogues from Fig. 1 were further investigated in living mammalian cells (Fig. 2).

Figure 2(a) highlights the high luciferase selectivity of the CTZ analogues selected from Fig. 1. The percentages (%) indicate the relative optical intensities of each substrate to the maximal value according to luciferases.

The live-cell images and time-course of bioluminescence developed by the key CTZ analogues were determined with COS-7 cells expressing various marine or beetle luciferases (Fig. 2(b)).

COS-7 cells grown in 6-channel microslides (μ-Slide VI^0.4^, ibidi) were transiently transfected with pcDNA 3.1(+) vector encoding GLuc, RLuc8.6-535, ALuc16, FLuc, CLuc, and CBgreen. Forty-eight hours after incubation, the cells in each channel were rinsed once with PBS and simultaneously bathed with 60 μL of native coelenterazine (nCTZ), 6etOH-CTZ, 6piOH-2H-CTZ, or 6piOH-CTZ dissolved in an assay buffer (final concentration: 10^−4^ M, Promega), using a multichannel pipet (Gilson). The microslides were immediately transferred to the dark chamber of the LAS-4000 (FujiFilm) and the corresponding optical intensities from the microslides were monitored every 5 minutes with a 4-minute integration mode after the substrate injection. The time-courses of the optical intensities from living COS-7 cells were: (i) measured time and area, i.e., the unit is RLU/sec/mm^2^, and (ii) normalized by the percentage of the maximal intensity (%) (Fig. 2(b)) using the specific image analyzing software, Multi Gauge v3.1 (Suppl. Fig. 5(a)).

### Highly specific illumination of hormonal activities of a ligand with mixed single-chain probes emitting almost superimposed optical spectra

The advantages of the luciferase-specific CTZ analogues were demonstrated with a unique multiplex assay system comprising two single-chain probes with almost superimposed optical spectra (Fig. 3).

For the experiment, we coexpressed two independent “molecular strain” probes, “TP2.4”^11^ and “ERS”^10^, which carry full-length ALuc23 and RLuc8, respectively (Fig. 3(a)). The full-length ALuc23 and RLuc8 are sandwiched between FRB and FKBP or between the ER LBD and the SH2 domain of *v*-Src, respectively. If the pair of proteins engage in an intramolecular protein‒protein interaction (PPI) via a ligand, the PPI applies molecular strain to the sandwiched full-length luciferase. The tensed luciferase is designed to enhance the optical intensities in a ligand-dependent manner.

pcDNA 3.1(+) vectors encoding TP2.4 and ERS were cotransfected into COS-7 cells in a 96-well optical bottom plate and incubated in a CO_2_ incubator for 16 h. The cotransfected cells in the microplate were partly stimulated with 10^−5^ M of dihydrotestosterone (DHT), cortisol, 17β-estradiol (E_2_), 4-hydroxytamoxifen (OHT), vehicle (0.1% DMSO) for 20 minutes, or partly with 10^−5^ M of rapamycin or vehicle (0.1% ethyl alcohol) for 4 h. After careful decantation of the medium, the cells were washed once with a PBS buffer and lysed with a lysis buffer (Promega) for 20 minutes. An aliquot of the lysates (10 μL) was transferred into a 1.6 mL microtube and mixed with 50 μL of 6etOH-CTZ, 6piOH-2H-CTZ, or D-luciferin, dissolved in Promega’s assay buffer. The corresponding optical intensities were immediately determined for 5 s with a luminometer (GloMax 20/20n, Promega).

## Electronic supplementary material

Suppl. Information
