# Supplementary material for: Luciferase-Specific Coelenterazine Analogues for Optical Contamination-Free Bioassays
Source: Sci Rep. 2017 Apr 19;7:908. doi: 10.1038/s41598-017-00955-6 (PMC5430434; doi:10.1038/s41598-017-00955-6)
Supplement: Supplementary file 1 — Suppl. Information [file 41598_2017_955_MOESM1_ESM.pdf]

Supplementary Information

Luciferase-Specific Coelenterazine Analogues for Optical Contamination-Free Bioassays

Ryo Nishihara,[1] Masahiro Abe,[1] Shigeru Nishiyama,[1] Daniel Citterio,[1] Koji Suzuki,[1] Sung Bae Kim,[2,*]

1. Department of Applied Chemistry, Faculty of Science and Technology, Keio University, 3-14-1 Hiyoshi, Kohoku-ku, Yokohama, Kanagawa 223-8522, Japan
2. Research Institute for Environmental Management Technology, National Institute of Advanced Industrial Science and Technology (AIST), 16-1 Onogawa, Tsukuba 305-8569, Japan

*** Corresponding author: kimu-sb@aist.go.jp**

Suppl. Figure 1. (A) The chemical structural characteristics between the grouped synthetic luciferins. The "X" and "Y" show any functional group at the C-6 and C-2 positions, respectively. Inset *a* shows the molecular structures of two marine luciferases, the crystal structure of *Renilla reniformis* luciferase 8 (RLuc8) with bound coelenteramide (the oxidized product of nCTZ) and a model of artificial luciferase 30 (ALuc30) in binding with nCTZ. **(B)** Various chemical structures of beetle and marine luciferins, including those newly synthesized in this study, are illustrated in groups (*i*−*v*). Inset *a* shows the bioluminescence spectra of representative beetle and marine luciferases. The green-filled area shows the spectrum of RLuc8.6-535.

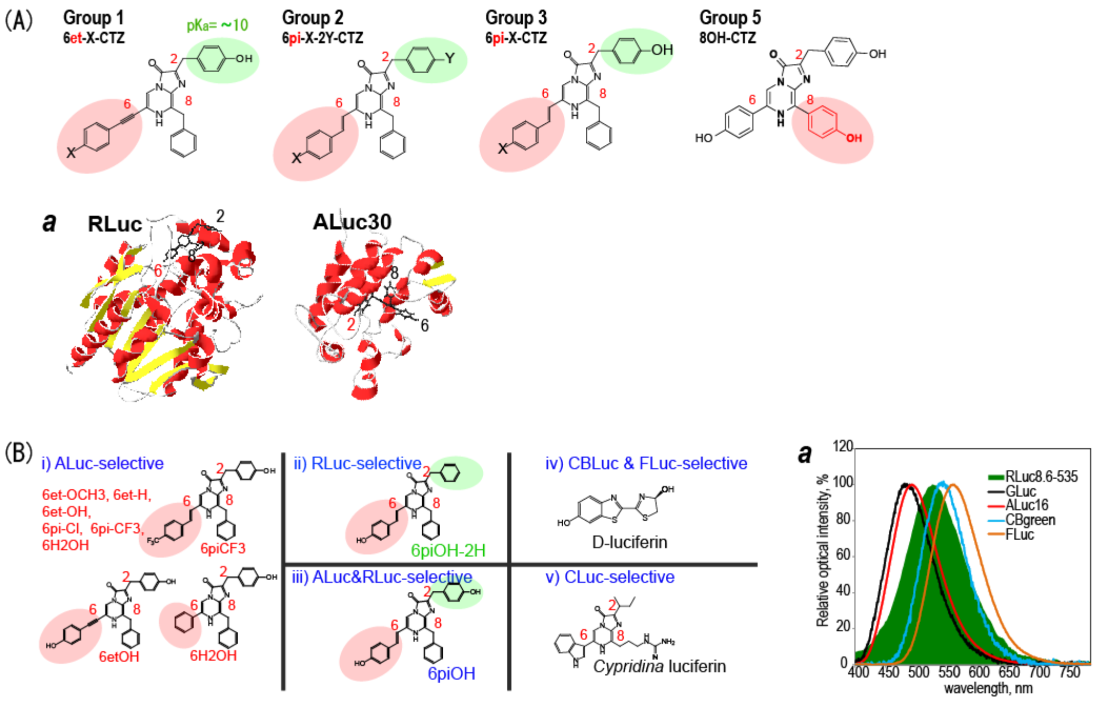

Suppl. Figure 2. Coelenteramide product (yellow) bound to the active site of RLuc8 (PDB ID: 2PSJ). The C-2 hydroxyphenyl forms hydrophobic interactions with Leu165 and Phe180 (cyan). Removal of the C-2 *para*-hydroxy group in the G3 analogue 6pi-OH-2H selectively activated RLuc8.6-535 perhaps by enabling an additional hydrophobic interaction with Met174 (yellow circle). However, the C-2 group is likely to possess more conformational freedom in the product than the substrate, and this structure may not represent a biologically relevant conformation. ALucs have very low sequence identity to RLuc (ca. 17%), and therefore most likely contain variant residues that interact with the C-2 group making ALucs sensitive to C-2 group substitutions. The C-8 phenylmethyl group is buried in a hydrophobic pocket consisting of Trp156 (green), Asp162 Cβ (not shown), Ile163, Ile166, Phe181, Val185 (salmon), Met174 and Phe180 (cyan). Chemical modifications to this group are likely to cause charge repulsion and steric hindrance. We suspect this hydrophobic pocket is highly conserved due to the abrogation of catalytic activity in all tested luciferases with the G5 analogue containing a C-8 *para*-hydroxy group (8phenol-CTZ). Increases in steric bulk to the C-6 hydroxyphenyl that produce a kink (G2 and G3 analogues) appear to be accommodated via orientation into free space deep in the active site pocket (magenta rectangle). The rigid increase in steric bulk associated with an ethynyl moiety (G1 analogues) might cause a steric clash with the putative catalytic triad (Asp120, Glu144 and His285) (blue ellipse). It should be noted that the putative catalytic triad is not in the immediate proximity of the imadazopyrazine backbone, so it is not clear if catalysis could occur in this conformation. It is possible that the substrate inserts deeper into the active site pocket, and that the 2PSJ crystal structure captured the coelenteramide product during its exit from the pocket.

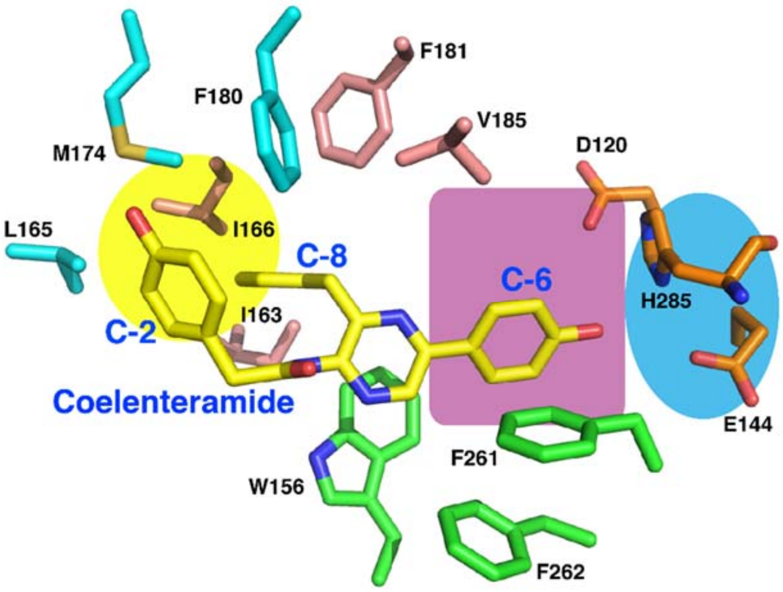

Suppl. Figure 3. Synthesis scheme for the synthetic coelenterazine analogues (6pi-CTZ series).

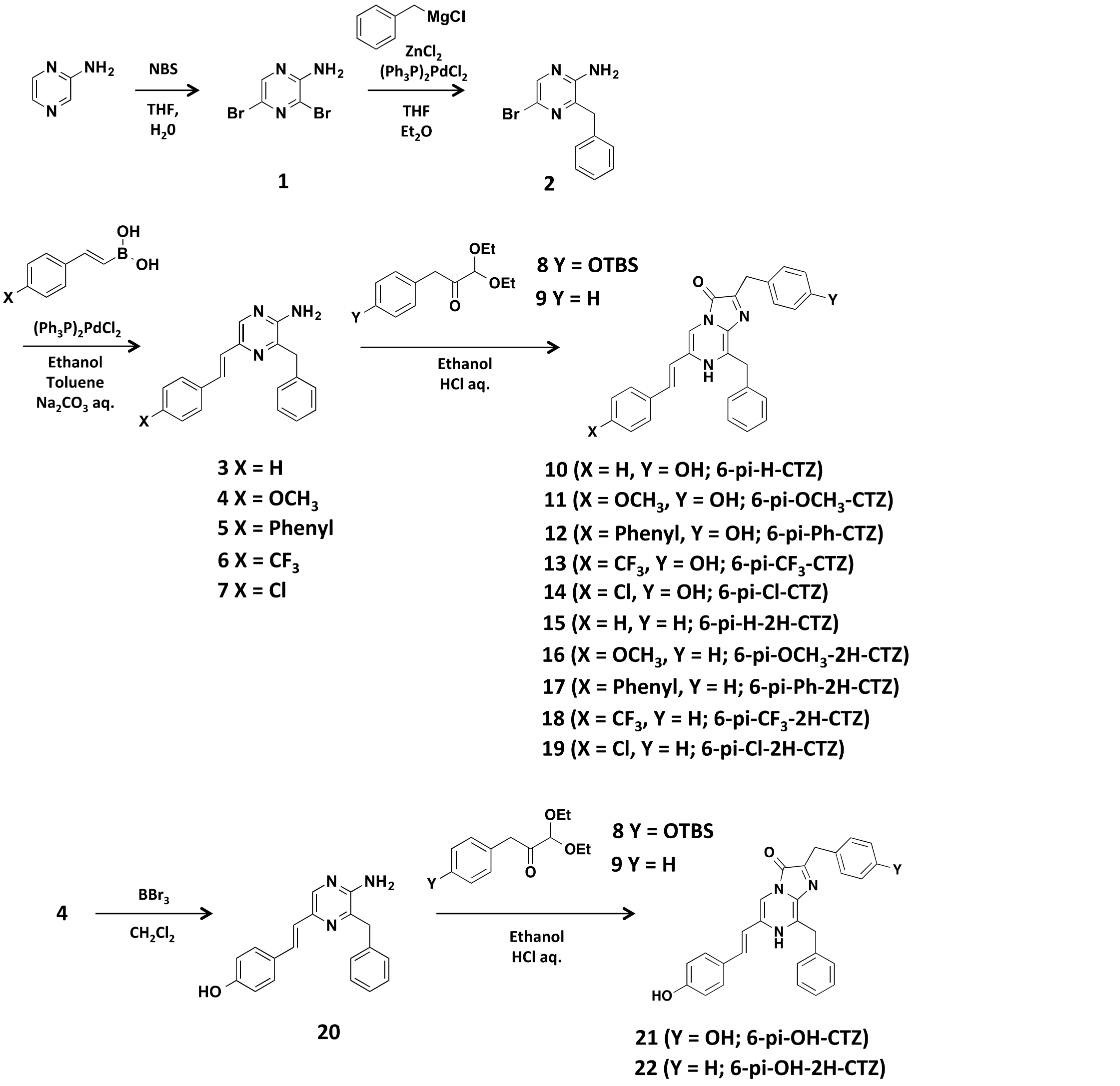

Compounds **1**, **2**, **3**, **4**, **5**, **8, 9**, **10**, **12**, **20** and **21** were synthesized based on reported procedures[9, 1-3].

Synthesis of ***(E)*-3-benzyl-5-(4-(trifluoromethyl)styryl)pyrazin-2-amine** (**6**)

3-Benzyl-5-bromopyrazine-2-amine (**2**) (250 mg, 0.95 mmol, 1 eq.) and (*E*)-(4-trifluoromethyl)styryl)boronic acid (328.3 mg, 1.52 mmol, 1.6 eq.) were dissolved in toluene (13.9 ml) and stirred at room temperature. Ethanol (2.1 ml) and 1 M Na_2CO_3 aq. (5 ml) were added into the reaction mixture. After vacuum deaeration, a catalytic amount of

tetrakis(triphenylphosphine)palladium(0) was added into the solution and the mixture was deaerated again and stirred for 11 hours at 100 °C. After cooling to room temperature, the solution was filtered through a Celite pad to remove the palladium catalyst. The solution was extracted with ethyl acetate, and the brown organic phase was washed with water and brine, dried over Na_2SO_4 and evaporated. The resulting residue was purified by flash chromatography (silica gel, eluent composition: n-hexane / ethyl acetate = 80/20 to 50/50), affording (*E*)-3-benzyl-5-(4-trifluoromethyl)styryl)pyrazine-2-amine (**6**) as a yellow solid (179.0 mg, 53%).

^{1}H-NMR (500 MHz, $CDCl_3$): δ (ppm) = 7.96 (s, 1H), 7.22-7.46 (m, 10H), 7.27 (d, *J* = 15.9 Hz, 1H), 4.59 (s, 2H), 4.13 (s, 2H). ^{13}C-NMR (125 MHz, $CDCl_3$): δ (ppm) = 41.49, 125.74, 125.77, 126.87, 127.22, 127.34, 127.99, 128.64, 129.22, 136.53, 140.18, 140.51, 140.77, 141.39, 152.21. HR-MS: m/z calcd for $C_{20}H_{16}F_3N_3$: 356.1375, found: 356.1357 $[M+H]^+$.

Synthesis of **(*E*)-3-benzyl-5-(4-chlorostyryl)pyrazin-2-amine** (**7**)

3-Benzyl-5-bromopyrazine-2-amine (**2**) (155 mg, 0.57 mmol, 1 eq.) and (*E*)-(4-chlorostyryl)boronic acid (165.75 mg, 0.91 mmol, 1.6 eq.) were dissolved in toluene (10.7 ml) and stirred at room temperature. Ethanol (1.5 ml) and 1 M Na_2CO_3 aq. (3.6 ml) were added into the reaction mixture. After vacuum deaeration, a catalytic amount of tetrakis(triphenylphosphine)palladium(0) was added into the solution and the mixture was deaerated again and stirred for 13 hours at 100 °C. After cooling to room temperature, the solution was filtered through a Celite pad to remove the palladium catalyst. The solution was extracted with ethyl acetate, and the brown organic phase was washed with water and brine, dried over Na_2SO_4 and evaporated. The resulting residue was purified by flash chromatography (silica gel, eluent composition: n-hexane / ethyl acetate = 50/50), affording (*E*)-3-benzyl-5-(4-chlorostyryl)pyrazine-2-amine (**7**) as a yellow solid (151.6 mg, 82%).

^{1}H-NMR (500 MHz, $CDCl_3$): δ (ppm) = 8.01 (s, 1H), 7.49-7.25 (m, 11H), 7.06 (d, *J* = 16.04 Hz, 1H),

4.43 (s, 2H), 4.16 (s, 2H). ^{13}C-NMR (125 MHz, $CDCl_3$): δ (ppm) = 41.45, 125.39, 127.27, 127.97, 128.31, 128.62, 128.96, 129.17, 133.37, 135.78, 136.61, 139.74, 140.90, 141.25, 151.96. HR-MS: m/z calcd for $C_{19}H_{16}ClN_3$: 322.1111, found: 322.1096 $[M+H]^+$.

Synthesis of **(*E*)-8-benzyl-2-(4-hydroxybenzyl)-6-(4-methoxystyryl)imidazo[1,2-*a*]pyrazin-3(*7H*)-one** (**11**; **6piOMe-CTZ**)

(*E*)-3-benzyl-5-(4-methoxystyryl)pyrazin-2-amine (**4**) (30 mg, 0.09 mmol, 1 eq.) and ketoacetal (**8**) (66.64 mg, 0.18 mmol, 2 eq.) were dissolved in ethanol (2.0 ml) and H_2O (0.2 ml) and stirred at room temperature. After vacuum dearation, the solution was cooled to 0 ˚C and HCl (0.1 ml) was added under nitrogen flow. Once the solution reached room temperature, it was heated and stirred for 14 hours at 80 °C. The solvent was evaporated under vacuum and the crude compound was purified by semipreparative reversed-phase HPLC (eluent composition: MeOH / H_2O = 25/75 with 0.1% formic acid), affording (*E*)-8-benzyl-2-(4-hydroxybenzyl)-6-(4-methoxystyryl)imidazo[1,2-*a*]pyrazin-3(*7H*)-one (**11**) as a yellow solid (7.34 mg, 16%).

^{1}H-NMR (500 MHz, CD_3OD, $CDCl_3$): δ (ppm) = 7.51 (s, 1H), 7.42-7.14 (m, 11H), 6.88 (d, J = 7.16 Hz, 2H), 6.77 (s, 1H), 6.69 (d, J = 6.87 Hz, 2H), 4.37 (s, 2H), 4.03 (s, 2H), 3.78 (s, 3H). ^{13}C-NMR (125 MHz, $CDCl_3$): δ (ppm) = 54.42, 113.93, 114.88, 126.87, 127.87, 128.35, 128.46, 128.72, 129.51, 136.68, 155.68, 160.311. HR-MS: m/z calcd for $C_{29}H_{26}N_3O_3$: 464.1974, found: 468.1977 $[M+H]^+$.

Synthesis of **(*E*)-8-benzyl-2-(4-hydroxybenzyl)-6-(4-(trifluoromethyl)styryl)imidazo[1,2-*a*]pyrazin-3(*7H*)-one** (**13**; **6piCF$_3$-CTZ**)

(*E*)-3-benzyl-5-(4-(trifluoromethyl)styryl)pyrazin-2-amine (**6**) (34.2 mg, 0.09 mmol, 1 eq.) and ketoacetal (**8**) (67.85 mg, 0.19 mmol, 2 eq.) were dissolved in ethanol (2.0 ml) and H_2O (0.2 ml) and stirred at room temperature. After vacuum dearation, the solution was cooled to 0 ˚C and HCl (0.1 ml)

was added under nitrogen flow. Once the solution reached room temperature, it was heated and stirred for 6 hours at 80 °C. The solvent was evaporated under vacuum and the crude compound was purified by silica colum chromatography (eluent composition: n-hexane / ethyl acetate = 50/50 to ethyl acetate to ethyl acetate / methanol = 20 /1), affording (*E*)-8-benzyl-2-(4-hydroxybenzyl)-6-(4-(trifluoromethyl)styryl)imidazo[1,2-*a*]pyrazin-3(*7H*)-one (**13**) as a yellow solid (11.99 mg, 29%).

^{1}H-NMR (500 MHz, CD_3OD): δ (ppm) = 7.66-7.61 (m, 5H), 7.37-7.19 (m, 6H), 7.13 (d, *J* = 8.59 Hz, 2H), 7.06 (d, *J* = 16.32 Hz, 1H), 6.68 (d, *J* = 8.59 Hz, 2H), 4.37 (s, 2H), 4.02 (s, 2H). ^{13}C-NMR (125 MHz, CD_3OD): δ (ppm) = 33.35, 34.99, 111.55, 116.21, 123.57, 124.56, 126.71, 126.74, 127.96, 128.15, 129.48, 129.58, 129.71, 129.75, 129.92, 130.51, 130.69, 130.80, 130.95, 138.05, 141.44, 157.00. HR-MS: m/z calcd for $C_{29}H_{23}F_3N_3O_2$: 502.1742, found: 502.1767 $[M+H]^+$.

Synthesis of **(*E*)-8-benzyl-6-(4-chlorostyryl)-2-(4-hydroxybenzyl)imidazo[1,2-*a*]pyrazin-3(*7H*)-one** (**14; 6piCl-CTZ**)

(*E*)-3-benzyl-5-(4-chlorostyryl)pyrazin-2-amine (**7**) (40 mg, 0.12 mmol, 1 eq.) and ketoacetal (**8**) (87.63 mg, 0.24 mmol, 2 eq.) were dissolved in ethanol (3.0 ml) and H_2O (0.3 ml) and stirred at room temperature. After vacuum dearation, the solution was cooled to 0 ˚C and HCl (0.2 ml) was added under nitrogen flow. Once the solution reached room temperature, it was heated and stirred for 4 hours at 80 °C. The solvent was evaporated under vacuum and the crude compound was purified by silica colum chromatography (eluent composition: dichloromethane / methanol = 20/1), affording (*E*)-8-benzyl-6-(4-chlorosyryl)-2-(4-hydroxybenzyl)imidazo[1,2-*a*]pyrazin-3(*7H*)-one (**14**) as a yellow solid (22.97 mg, 39%).

^{1}H-NMR (500 MHz, CD_3OD, $CDCl_3$): δ (ppm) = 7.48 (s, 1H), 7.42 (d, *J* = 8.59 Hz, 2H), 7.38-7.22 (m, 7H), 7.20 (d, *J* = 8.59 Hz, 2H), 7.12 (d, 1H), 6.79 (d, *J* = 16.32 Hz, 1H), 6.74 (d, *J* = 8.59 Hz, 2H), 4.36 (s, 2H), 4.08 (s, 2H). ^{13}C-NMR (125 MHz, CD_3OD, $CDCl_3$): δ (ppm) = 33.18, 109.75, 109.84,

115.70, 127.64, 128.32, 129.09, 129.17, 129.41, 129.65, 130.29, 134.60, 136.84, 155.83. HR-MS: m/z calcd for $C_{28}H_{23}ClN_3O_2$: 468.1479, found: 468.1458 $[M+H]^+$.

Synthesis of **(*E*)-2,8-dibenzyl-6-styrylimidazo[1,2-*a*]pyrazin-3(*7H*)-one (15; 6piH-2H-CTZ)**

(*E*)-3-benzyl-5-styrylpyrazin-2-amine (**3**) (40.00 mg, 0.13 mmol, 1 eq.) and ketoacetal (**9**) (61.88 mg, 0.27 mmol, 2 eq.) were dissolved in ethanol (3.0 ml) and H_2O (0.3 ml) and stirred at room temperature. After vacuum dearation, the solution was cooled to 0 ˚C and HCl (0.2 ml) was added under nitrogen flow. Once the solution reached room temperature, it was heated and stirred for 3.5 hours at 80 °C. The solvent was evaporated under vacuum and the crude compound was purified by silica colum chromatography (eluent composition: dichloromethane / methanol = 20/1), affording (*E*)-2,8-dibenzyl-6-styrylimidazo[1,2-*a*]pyrazin-3(*7H*)-one (**15**) as a yellow solid (32.06 mg, 44%)

^{1}H-NMR (500 MHz, CD_3OD): δ (ppm) = 7.48-7.15 (m, 17H), 6.80 (d, J = 16.61 Hz, 1H), 4.36 (s, 2H), 4.17 (s, 2H). ^{13}C-NMR (125 MHz, CD_3OD, $CDCl_3$): δ (ppm) = 33.92, 34.17, 109.50, 109.68, 119.02, 119.50, 126.90, 127.20, 127.55, 127.76, 128.96, 129.12, 129.22, 129.28, 129.35, 129.38, 131.41, 136.55, 136.99, 138.88, 153.24. HR-MS: m/z calcd for $C_{28}H_{24}N_3O$: 418.1919, found: 418.1910 $[M+H]^+$.

Synthesis of **(*E*)-2,8-dibenzyl-6-(4-methoxystyryl)imidazo[1,2-*a*]pyrazin-3(*7H*)-one (16; 6piOMe-2H-CTZ)**

(*E*)-3-benzyl-5-(4-methoxystyryl)pyrazin-2-amine (**4**) (42.85 mg, 0.13 mmol, 1 eq.) and ketoacetal (**9**) (60.01 mg, 0.27 mmol, 2 eq.) were dissolved in ethanol (3.0 ml) and H_2O (0.3 ml) and stirred at room temperature. After vacuum dearation, the solution was cooled to 0 ˚C and HCl (0.2 ml) was added under nitrogen flow. Once the solution reached room temperature, it was heated and stirred for

15 hours at 80 °C. The solvent was evaporated under vacuum and the crude compound was purified by silica colum chromatography (eluent composition: dichloromethane / methanol = 20/1), affording (*E*)-2,8-dibenzyl-6-(4-methoxystyryl)imidazo[1,2-*a*]pyrazin-3(*7H*)-one (**16**) as a yellow solid (31.4 mg, 51%).

^{1}H-NMR (500 MHz, CD_3OD, $CDCl_3$): δ (ppm) = 7.50 (s, 1H), 7.43 (d, *J* = 8.59 Hz, 2H), 7.39-7.15 (m, 11H), 6.90 (d, *J* = 8.88 Hz, 2H), 6.72 (d, *J* = 16.32 Hz, 1H), 4.38 (s, 2H), 4.17 (s, 2H), 3.33 (s, 3H). ^{13}C-NMR (125 MHz, CD_3OD, $CDCl_3$): δ (ppm) = 30.35, 34.22, 55.66, 109.20, 114.97, 127.04, 127.90, 128.73, 128.81, 129.03, 129.12, 129.36, 129.44, 129.52, 129.58, 131.39, 131.97, 137.28, 139.18. HR-MS: m/z calcd for $C_{29}H_{26}N_3O_2$: 448.2025, found: 448.2035 $[M+H]^+$.

Synthesis of **(*E*)-6-(2-([1,1'-biphenyl]-4-yl)vinyl)-2,8-dibenzylimidazo[1,2-*a*]pyrazin-3(*7H*)-one (17; 6piPh-2H-CTZ**)

(*E*)-5-(2-([1,1'-biphenyl]-4-yl)vinyl)-3-benzylpyrazin-2-amine (**5**) (30.00 mg, 0.11 mmol, 1 eq.) and ketoacetal (**9**) (50.49 mg, 0.22 mmol, 2 eq.) were dissolved in ethanol (2.0 ml) and H_2O (0.2 ml) and stirred at room temperature. After vacuum dearation, the solution was cooled to 0 °C and HCl (0.1 ml) was added under nitrogen flow. Once the solution reached room temperature, it was heated and stirred for 6 hours at 80 °C. The solvent was evaporated under vacuum and the crude compound was purified by silica colum chromatography (eluent composition: ethyl acetate), affording (*E*)-6-(2-([1,1'-biphenyl]-4-yl)vinyl)-2,8-dibenzylimidazo[1,2-*a*]pyrazin-3(*7H*)-one (17) as yellow solid (13.36 mg, 24%)

^{1}H-NMR (500 MHz, CD_3OD, $CDCl_3$): δ (ppm) = 7.61-7.16 (m, 21H), 6.89 (d, *J* = 16.32 Hz, 1H), 4.39 (s, 2H), 4.16 (s, 2H). ^{13}C-NMR (125 MHz, CD_3OD, $CDCl_3$): δ (ppm) = 34.17, 110.02, 127.08, 127.48, 127.93, 128.06, 128.24, 129.14, 129.39, 129.47, 129.53, 131.16, 135.88, 137.25, 139.11, 141.12, 142.13. HR-MS: m/z calcd for $C_{34}H_{27}N_3O$: 494.2232, found: 494.2220 $[M+H]^+$.

Synthesis of **(*E*)-2,8-dibenzyl-6-(4-(trifluoromethyl)styryl)imidazo[1,2-*a*]pyrazin-3(*7H*)-one (18; 6piCF$_3$-2H-CTZ**)

(*E*)-3-benzyl-5-(4-(trifluoromethyl)styryl)pyrazin-2-amine (**6**) (30.00 mg, 0.08 mmol, 1 eq.) and ketoacetal (**9**) (35.56 mg, 0.16 mmol, 2 eq.) were dissolved in ethanol (2.0 ml) and H_2O (0.2 ml) and stirred at room temperature. After vacuum dearation, the solution was cooled to 0 ˚C and HCl (0.1 ml) was added under nitrogen flow. Once the solution reached room temperature, it was heated and stirred for 4 hours at 80 ℃. The solvent was evaporated under vacuum and the crude compound was purified by silica colum chromatography (eluent composition: chloroform / methanol = 20/1), affording (*E*)-2,8-dibenzyl-6-(4-(trifluoromethyl)styryl)imidazo[1,2-*a*]pyrazin-3(*7H*)-one (**18**) as yellow solid (22.58 mg, 58%)

^{1}H-NMR (500 MHz, $CDCl_3$): δ (ppm) = 7.62-6.95 (m, 17H), 6.57 (s, 1H), 4.38 (s, 2H), 4.10 (s, 2H). ^{13}C-NMR (125 MHz, $CDCl_3$): δ (ppm) = 34.45, 109/96, 123.01, 125.17, 125.81, 126.49, 126.72, 127.22, 127.33, 127.48, 128.28, 128.49, 128.64, 128.91, 129.14, 129.28, 129.91, 130.17, 136.18, 138.16, 139.22. HR-MS: m/z calcd for $C_{29}H_{23}F_3N_3O$: 486.1793, found: 486.1764 $[M+H]^+$.

Synthesis of **(*E*)-2,8-dibenzyl-6-(4-chlorostyryl)imidazo[1,2-*a*]pyrazin-3(*7H*)-one (19; 6piCl-2H-CTZ)**

(*E*)-3-benzyl-5-(4-chlorostyryl)pyrazin-2-amine (**7**) (40.21 mg, 0.12 mmol, 1 eq.) and ketoacetal (**9**) (55.54 mg, 0.24 mmol, 2 eq.) were dissolved in ethanol (3.0 ml) and H_2O (0.3 ml) and stirred at room temperature. After vacuum dearation, the solution was cooled to 0 ˚C and HCl (0.2 ml) was added under nitrogen flow. Once the solution reached room temperature, it was heated and stirred for 3.5 hours at 80 ℃. The solvent was evaporated under vacuum and the crude compound was purified

by silica colum chromatography (eluent composition: dichloromethane / methanol = 20/1), affording (*E*)-2,8-dibenzyl-6-(4-chlorostyryl)imidazo[1,2-*a*]pyrazin-3(*7H*)-one (**19**) as yellow solid (15.32 mg, 27 %)

^{1}H-NMR (500 MHz, CD_3OD, $CDCl_3$): δ (ppm) = 7.54 (s, 1H), 7.44 (d, J = 8.31 Hz, 2H), 7.39-7.16 (m, 13H), 6.85 (d, J = 16.32 Hz, 1H), 4.38 (s, 2H), 4.17 (s, 2H). ^{13}C-NMR (125 MHz, CD_3OD, $CDCl_3$): δ (ppm) = 14.35, 34.23, 110.20, 120.32, 127.00, 127.43, 127.85, 128.61, 129.07, 129.31, 129.38, 129.47, 129.61, 130.10, 134.83, 135.47, 137.15, 139.02. HR-MS: m/z calcd for $C_{28}H_{22}ClN_3O$: 452.1530, found: 452.1510 $[M+H]^+$.

Synthesis of **(*E*)-2,8-dibenzyl-6-(4-hydroxystyryl)imidazo[1,2-*a*]pyrazin-3(*7H*)-one** (**22; 6piOH-2H-CTZ**)

(E)-4-(2-(5-Amino-6-benzylpyrazin-2-yl)vinyl)phenol (**20**) (40 mg, 0.13 mmol, 1 eq.) and ketoacetal (**9**) (58 mg, 0.26 mmol, 2 eq.) were dissolved in ethanol (2.0 ml) and H_2O (0.2 ml) and stirred at room temperature. After vacuum dearation, the solution was cooled to 0 ˚C and HCl (0.1 ml) was added under nitrogen flow. Once the solution reached room temperature, it was heated and stirred for 5 hours at 80 ℃. The solvent was evaporated under vacuum and the crude compound was purified by silica colum chromatography (eluent composition: n-hexane / ethyl acetate = 50/50 to ethyl acetate to ethyl acetate / methanol = 20 /1), affording (*E*)-2,8-dibenzyl-6-(4-hydroxystyryl) imidazo[1,2-*a*]pyrazin-3(*7H*)-one (**22**) as a yellow solid (27.83 mg, 49%).

^{1}H-NMR (500 MHz, CD_3OD): δ (ppm) = 7.42 (s, 1H), 7.35-7.10 (m, 12H), 7.05 (d, J = 16.6 Hz, 1H), 6.73 (d, J = 8.6 Hz, 2H), 6.56 (d, J = 16.6 Hz, 2H), 4.31 (s, 2H), 4.08 (s, 2H) ^{13}C-NMR (125 MHz, CD_3OD): δ (ppm) = 34.3, 34.5, 109.3, 116.6, 127.3, 128.1, 128.8, 129.2, 129.4, 129.6, 129.7, 129.8, 132.0, 137.9, 139.7, 159.3. HR-MS: m/z calcd for $C_{28}H_{24}N_3O_2$: 434.1869, found: 434.1857 $[M+H]^+$.

Suppl. Figure 4. Synthesis scheme for the synthetic coelenterazine analogues (6-ethynyl-CTZ series).

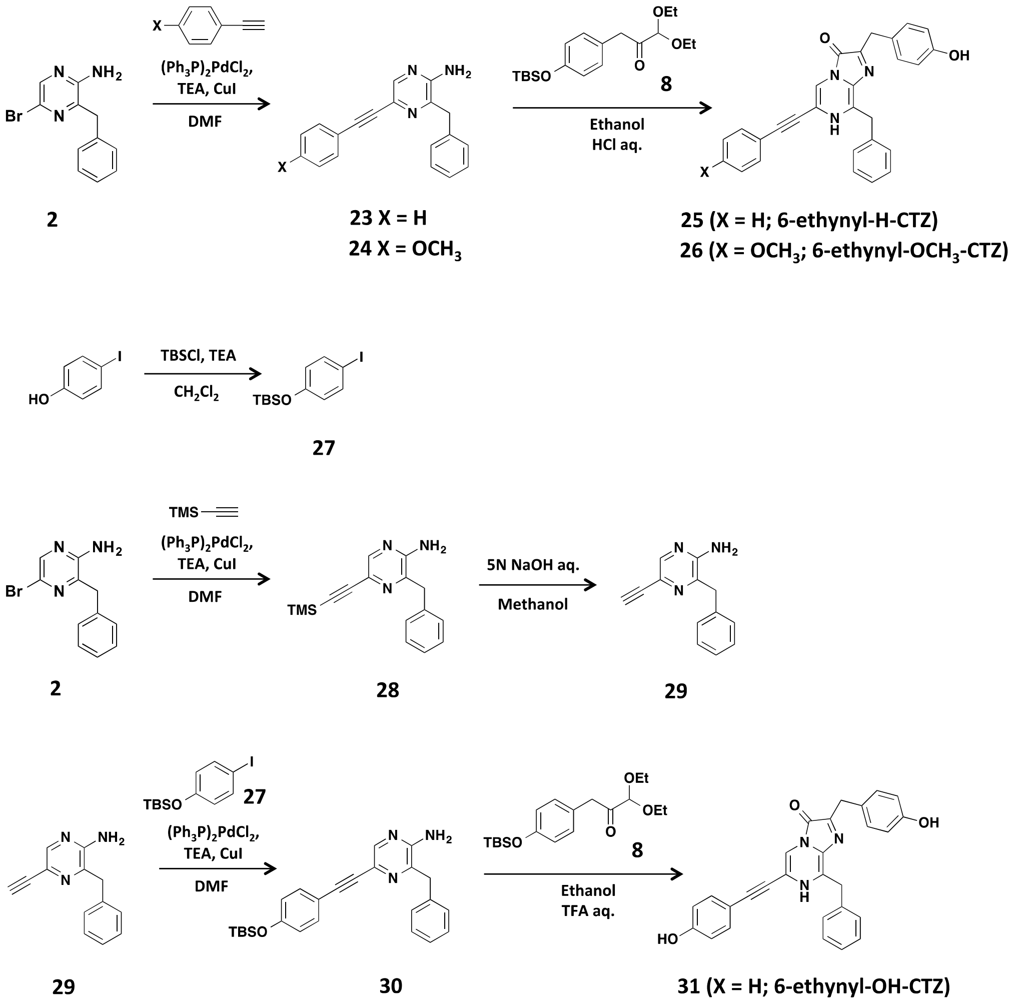

Compound **27, 28 and 29** were synthesized based on reported procedures [4,5].

Synthesis of **3-benzyl-5-(phenylethynyl)pyrazin-2-amine (23)**

3-Benzyl-5-bromopyrazine-2-amine (**2**) (160.00 mg, 0.60 mmol, 1 eq.) was dissolved in DMF (12.8 ml) and TEA (1.0 ml) and stirred at room temperature. Phenylacetylene (0.20 ml, 1.82 mmol, 3 eq.) was added into the reaction mixture. After vacuum deaeration, a catalytic amount of tetrakis(triphenylphosphine)palladium(0) and CuI was added into the solution and the mixture was

deaerated again and stirred for 18 hours at 120 ℃. After cooling to room temperature, the solution was filtered through a Celite pad to remove the catalysts. The solution was extracted with ethyl acetate, and the organic phase was washed with water and brine, dried over Na_2SO_4 and evaporated. The resulting residue was purified by flash chromatography (silica gel, eluent composition: n-hexane / ethyl acetate = 67/33), affording 3-benzyl-5-(phenylethynyl)pyrazin-2-amine (**23**) as a yellow solid (163.0 mg, 94%).

^{1}H-NMR (500 MHz, $CDCl_3$): δ (ppm) = 8.21 (s, 1H), 7.56-7.62 (m, 2H), 7.33-7.37 (m, 2H), 4.54 (s, 2H), 4.17 (s, 2H). ^{13}C-NMR (125 MHz, $CDCl_3$): δ (ppm) = 41.24, 86.54, 89.90, 122.54, 127.22, 128.36, 128.39, 128.55, 129.09, 131.73, 136.03, 141.17, 144.36, 151.68. HR-MS: m/z calcd for $C_{19}H_{15}N_3$: 286.1344, found: 286.1331 $[M+H]^+$.

Synthesis of **3-benzyl-5-((4-methoxyphenyl)ethynyl)pyrazin-2-amine (24)**

3-Benzyl-5-bromopyrazine-2-amine (**2**) (154.00 mg, 0.58 mmol, 1 eq.) was dissolved in DMF (12.0 ml) and TEA (1.0 ml) and stirred at room temperature. p-Ethynylanisole (0.23 ml, 1.72 mmol, 3 eq.) was added into the reaction mixture. After vacuum deaeration, a catalytic amount of tetrakis(triphenylphosphine)palladium(0) and CuI was added into the solution and the mixture was deaerated again and stirred for 14 hours at 120 ℃. After cooling to room temperature, the solution was filtered through a Celite pad to remove the catalysts. The solution was extracted with ethyl acetate, and the organic phase was washed with water and brine, dried over Na_2SO_4 and evaporated. The resulting residue was purified by flash chromatography (silica gel, eluent composition: n-hexane / ethyl acetate = 67/33), affording 3-benzyl-5-((4-methoxyphenyl)ethynyl)pyrazin-2-amine (24) as a yellow solid (181.0 mg, 98%).

^{1}H-NMR (500 MHz, $CDCl_3$): δ (ppm) = 8.18 (s, 1H), 7.53 (d, *J* = 8.4 Hz, 2H), 7.24-7.35 (m, 5H), 6.89 (d, *J* = 8.4 Hz, 2H), 4.50 (s, 2H), 4.16 (s, 2H), 3.84 (s, 3H). ^{13}C-NMR (125 MHz, $CDCl_3$): δ

(ppm) = 41.27, 55.25, 85.29, 90.00, 113.90, 113.97, 114.61, 127.20, 128.42, 129.08, 133.26, 136.12, 141.10, 141.14, 151.48, 159.83. HR-MS: m/z calcd for $C_{20}H_{17}N_3O$: 316.1450, found: 316.1442 $[M+H]^+$.

Synthesis of **8-benzyl-2-(4-hydroxybenzyl)-6-(phenylethynyl)imidazo[1,2-*a*]pyrazin-3(*7H*)-one (25, 6etH-CTZ)**

3-benzyl-5-(phenylethynyl)pyrazin-2-amine (**23**) (49.9 mg, 0.17 mmol, 1 eq.) and ketoacetal (**8**) (128.0 mg, 0.36 mmol, 2.1 eq.) were dissolved in ethanol (3.3 ml) and H_2O (0.3 ml) and stirred at room temperature. After vacuum dearation, the solution was cooled to 0 ˚C and TFA (0.2 ml) was added under nitrogen flow. Once the solution reached room temperature, it was heated and stirred for 4 hours at 70 ℃. The solvent was evaporated under vacuum and the crude compound was purified by silica column chromatography (eluent composition: dichloromethane / methanol = 30/1 to 15/1) and reversed phase PLC (eluent composition: acetonitrile / H_2O = 50/50), affording 8-benzyl-2-(4-hydroxybenzyl)-6-(phenylethynyl)imidazo[1,2-*a*]pyrazin-3(*7H*)-one (**25**) as yellow solid (11.9 mg, 16%).

^{1}H-NMR (500 MHz, CD_3OD): δ (ppm) = 7.69 (s, 1H), 7.23-7.31 (m, 5H), 7.36-7.55 (m, 5H), 7.16 (d, J = 8.2 Hz, 2H), 6.69 (d, J = 8.2 Hz, 2H), 4.31 (s, 2H), 4.06 (s, 2H). ^{13}C-NMR (125 MHz, CD_3OD, $CDCl_3$): δ (ppm) = 33.45, 35.17, 82.07, 93.55, 115.50, 116.04, 122.64, 127.41, 128.00, 129.37, 129.50, 129.53, 129.70, 130.27, 130.27, 130.68, 132.49, 137.73, 156.55. HR-MS: m/z calcd for $C_{28}H_{21}N_3O_2$: 430.1556, found: 430.1557 $[M-H]^-$.

Synthesis of **8-benzyl-2-(4-hydroxybenzyl)-6-((4-methoxyphenyl)ethynyl)imidazo[1,2-*a*]pyrazin-3(*7H*)-one (26, 6etOMe-CTZ)**

3-benzyl-5-((4-methoxyphenyl)ethynyl)pyrazin-2-amine (**24**) (29.7 mg, 0.09 mmol, 1 eq.) and

ketoacetal (**8**) (67.1 mg, 0.19 mmol, 2.1 eq.) were dissolved in ethanol (2.0 ml) and H_2O (0.1 ml) and stirred at room temperature. After vacuum dearation, the solution was cooled to 0 ˚C and TFA (0.2 ml) was added under nitrogen flow. Once the solution reached room temperature, it was heated and stirred for 4.5 hours at 70 ℃. The solvent was evaporated under vacuum and the crude compound was purified by silica column chromatography (eluent composition: dichloromethane / methanol = 30/1 to 20/1) and reversed phase PLC (eluent composition: acetonitrile / H_2O = 50/50), affording 8-benzyl-2-(4-hydroxybenzyl)-6-((4-methoxyphenyl)ethynyl)imidazo[1,2-*a*]pyrazin-3(*7H*)-one (**26**) as yellow solid (7.96 mg, 18%).

^{1}H-NMR (500 MHz, CD_3OD): δ (ppm) = 7.63 (s, 1H), 7.48 (d, J = 8.8 Hz, 2H), 7.23-7.37 (m, 5H), 7.15 (d, J = 8.5 Hz, 2H), 6.94 (d, J = 8.8 Hz, 2H), 6.69 (d, J = 8.5 Hz, 2H), 4.31 (s, 2H), 4.06 (s, 2H), 3.82 (s, 3H). ^{13}C-NMR (100 MHz, CD_3OD): δ (ppm) = 33.63, 34.86, 80.27, 94.45, 114.48, 114.91, 115.33, 115.61, 116.19, 127.48, 128.23, 129.75, 129.81, 130.64, 130.85, 132.73, 134.31, 137.83, 156.97, 162.13. HR-MS: m/z calcd for $C_{29}H_{23}N_3O_3$: 460.1661, found: 460.1673 $[M-H]^-$.

Synthesis of **3-benzyl-5-((trimethylsilyl)ethynyl)pyrazin-2-amine (28)**

3-Benzyl-5-bromopyrazine-2-amine (**2**) (267.00 mg, 1.01 mmol, 1 eq.) was dissolved in DMF (12.0 ml) and TEA (1.5 ml) and stirred at room temperature. Ethynyltrimethylsilane (0.57 ml, 4.03 mmol, 4 eq.) was added into the reaction mixture. After vacuum deaeration, a catalytic amount of tetrakis(triphenylphosphine)palladium(0) and CuI was added into the solution and the mixture was deaerated again and stirred for 20.5 hours at 120 ℃. After cooling to room temperature, the solution was filtered through a Celite pad to remove the catalysts. The solution was extracted with ethyl acetate, and the organic phase was washed with water and brine, dried over Na_2SO_4 and evaporated. The resulting residue was purified by flash chromatography (silica gel, eluent composition: n-hexane / ethyl acetate = 67/33), affording 3-benzyl-5-((trimethylsilyl)ethynyl)pyrazin-2-amine (28) as a

yellow solid (188.7 mg, 66%).^{1}H-NMR (400 MHz, $CDCl_3$): δ (ppm) = 8.13 (s, 1H), 7.20-7.32 (m, 5H), 4.54 (s, 2H), 4.13 (s, 2H), 0.28 (s, 9H).

Synthesis of **3-benzyl-5-ethynylpyrazin-2-amine (29)**

3-benzyl-5-((trimethylsilyl)ethynyl)pyrazin-2-amine (**28**) (99.9 mg, 0.35 mmol, 1 eq.) was dissolved in methanol (5.0 ml) and stirred at room temperature. 5N NaOH aq. (0.25 ml, 1.00 mmol, 2.81 eq.) was added into the reaction mixture and stirred at room temperature for 3 hours. 1N HCl aq. was added into the reaction to quench and evaporated to dryness. The residue was dissolved and extracted with ethyl acetate, and the organic layer was washed with water and brine, dried over Na_2SO_4, and evaporated. The resulting residue was purified by flash chromatography (silica gel, eluent composition: n-hexane / ethyl acetate = 67/33), affording 3-benzyl-5-ethynylpyrazin-2-amine (**29**) as a yellow solid (66.8 mg, 90%).

^{1}H-NMR (400 MHz, $CDCl_3$): δ (ppm) = 8.14 (s, 1H), 7.22-7.33 (m, 5H), 4.56 (s, 2H), 4.13 (s, 2H), 3.21 (s, 1H). HR-MS: m/z calcd for $C_{13}H_{11}N_3$: 210.1031, found: 210.1029 $[M+H]^+$.

Synthesis of **3-benzyl-5-((4-((*tert*-butyldimethylsilyl)oxy)phenyl)ethynyl)pyrazin-2-amine (30)**

3-benzyl-5-ethynylpyrazin-2-amine (**29**) (91.00 mg, 0.27 mmol, 1 eq.) and tert-butyl(4-iodophenoxy)dimethylsilane (**27**) (56.9 mg, 0.27 mmol, 1 eq.) were dissolved in DMF (10.0 ml) and TEA (3.0 ml) and stirred at room temperature. After vacuum deaeration, a catalytic amount of tetrakis(triphenylphosphine)palladium(0) and CuI was added into the solution and the mixture was deaerated again and stirred for 2 hours at room temperature. The solution was filtered through a Celite pad to remove the catalysts. The solution was extracted with ethyl acetate, and the organic phase was washed with water and brine, dried over Na_2SO_4 and evaporated. The resulting residue was purified by flash chromatography (silica gel, eluent composition: n-hexane / ethyl acetate = 70/30 to 60/40

to 55/45), affording 3-benzyl-5-((4-((*tert*-butyldimethylsilyl)oxy)phenyl)ethynyl)pyrazin-2-amine (**30**) as a yellow solid (65.1 mg, 58%).

^{1}H-NMR (400 MHz, $CDCl_3$): δ (ppm) = 8.17 (s, 1H), 7.48 (d, *J* = 8.8 Hz, 2H), 7.26-7.49 (m, 5H), 6.82 (d, *J* = 8.8 Hz, 2H), 4.50 (s, 2H), 4.16 (s, 2H), 0.99 (s, 9H), 0.22 (s, 6H). ^{13}C-NMR (100 MHz, $CDCl_3$): δ (ppm) = -4.43, 18.19, 25.60, 41.30, 85.40, 90.10, 115.25, 120.17, 127.23, 128.43, 128.82, 129.11, 133.26, 136.10, 114.16, 144.14, 151.45, 156.25. HR-MS: m/z calcd for $C_{25}H_{29}N_3OSi$: 416.2158, found: 416.2131 $[M+H]^+$.

Synthesis of **8-benzyl-2-(4-hydroxybenzyl)-6-((4-hydroxyphenyl)ethynyl)imidazo[1,2-*a*]pyrazin-3(*7H*)-one (31, 6etOH-CTZ)**

3-benzyl-5-((4-((*tert*-butyldimethylsilyl)oxy)phenyl)ethynyl)pyrazin-2-amine (**30**) (33.0 mg, 0.07 mmol, 1 eq.) and ketoacetal (**8**) (30.8 mg, 0.07 mmol, 2.1 eq.) were dissolved in ethanol (2.0 ml) and H_2O (0.2 ml) and stirred at room temperature. After vacuum dearation, the solution was cooled to 0 °C and TFA (0.3 ml) was added under nitrogen flow. Once the solution reached room temperature, it was heated and stirred for 3 hours at 80 ℃. The solvent was evaporated under vacuum and the crude compound was purified by silica column chromatography (eluent composition: chloroform / methanol = 20/1 to 10/1) and reversed phase PLC (eluent composition: methanol / H_2O = 80/20), affording 8-benzyl-2-(4-hydroxybenzyl)-6-((4-hydroxyphenyl)ethynyl)imidazo[1,2-*a*]pyrazin-3(*7H*)-one (**31**) as yellow solid (6.5 mg, 20%)

^{1}H-NMR (400 MHz, CD_3OD): δ (ppm) = 7.65 (s, 1H), 7.20-7.36 (m, 7H), 7.14 (d, *J* = 8.4 Hz, 2H), 6.78 (d, *J* = 8.8 Hz, 2H), 6.69 (d, *J* = 8.4 Hz, 2H), 4.30 (s, 2H), 4.05 (s, 2H). ^{13}C-NMR (150 MHz, CD_3OD): δ (ppm) = 33.71, 34.72, 79.47, 95.19, 113.09, 114.65, 116.24, 116.61, 116.76, 127.49, 128.30, 129.66, 129.81, 130.59, 130.89, 134.50, 137.78, 157.01, 160.32. HR-MS: m/z calcd for $C_{28}H_{21}N_3O_3$: 448.1661, found: 448.1678 $[M-H]^-$.

Suppl. Figure 5. Half-lives of the bioluminescence intensities of marine luciferases according to luciferins in live cells. **(A)** Time-course of the decay of the relative optical intensities of various luciferase─luciferin pairs. **(B)** Time course of the decay of the absolute optical intensities (RLU /sec/mm^2) of the 6etOH-CTZ─ALuc16 pair.

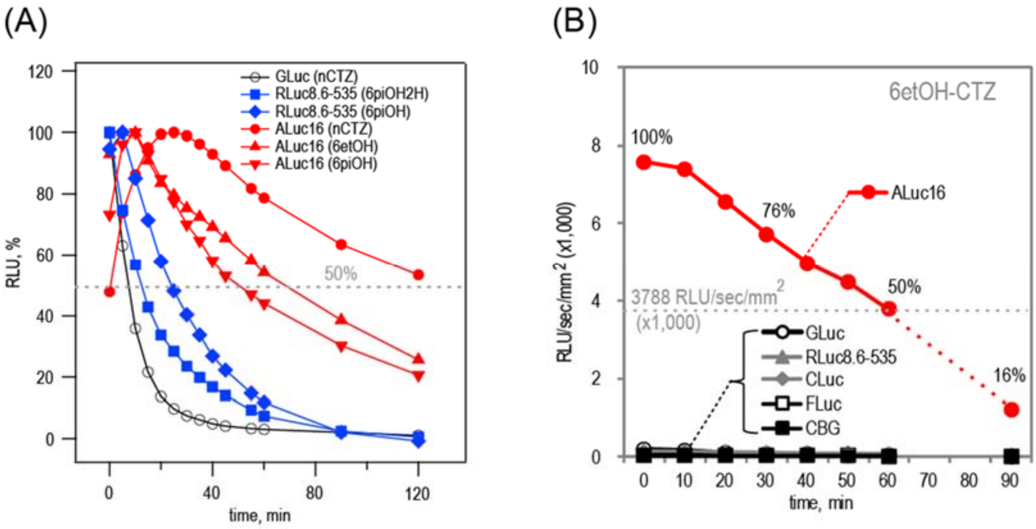

Suppl. Figure 6. pH-driven features of CTZ─luciferase activity. **(A)** pH-dominated optical variance of nCTZ with marine luciferases. **(B)** pH-driven optical variance of 6piOH-CTZ with marine luciferases. **(C)** The absolute optical intensities of (A) showing nCTZ─luciferase activities according to pH (RLU/sec/mm^2) (*n*=3).

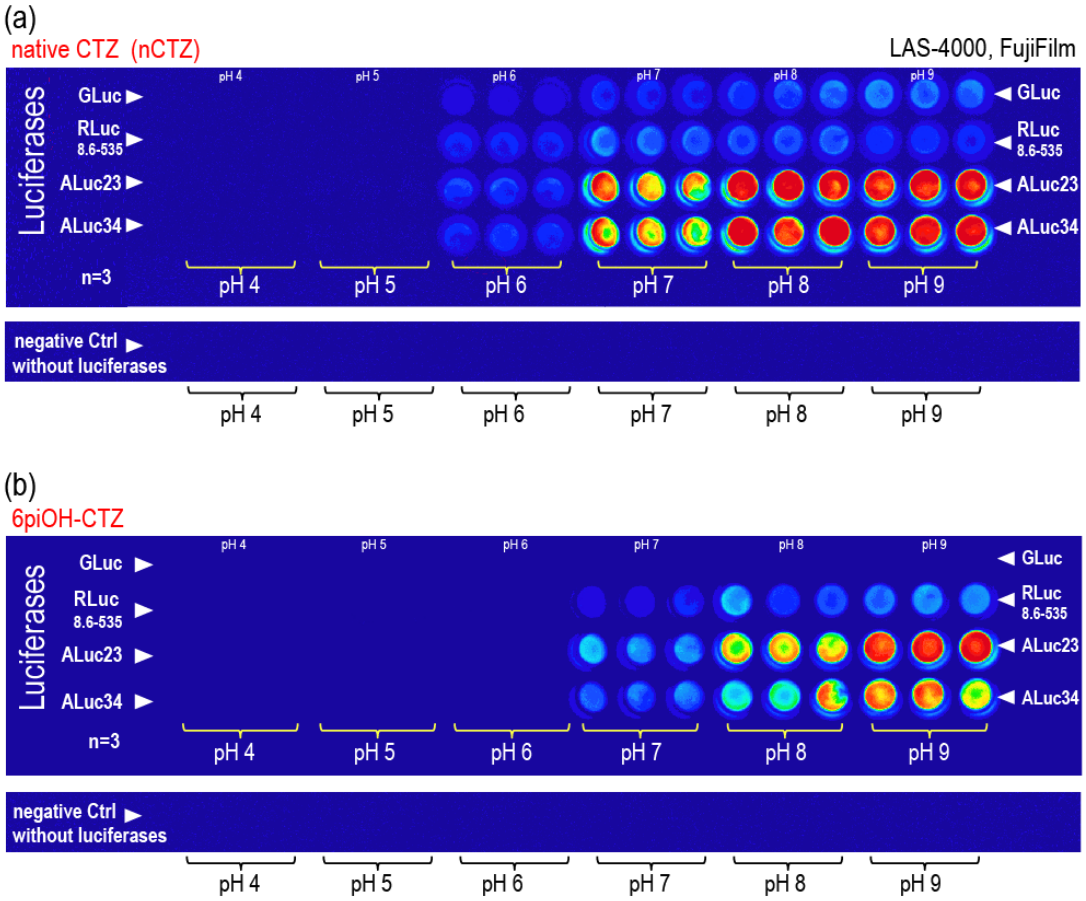

(c)

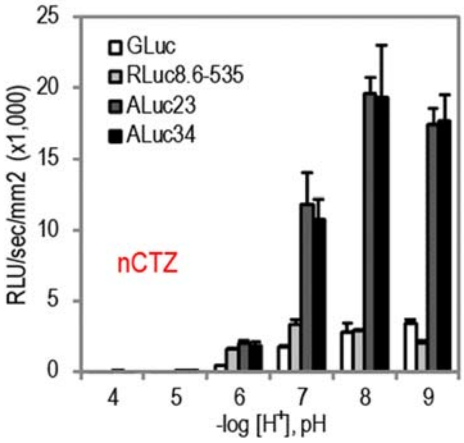
GLuc
RLuc8.6-535
ALuc23
ALuc34
nCTZ
RLU/sec/mm2 (x1,000)
0
5
10
15
20
25
4
5
6
7
8
9
-log [H+], pH

Suppl. Figure 7. pH-driven optical intensities of CTZ analogues with purified ALuc16. **(A)** The optical image of bioluminescence driven by varying pHs. Each CTZ analogue exhibited a distinctive pH preference with purified ALuc16. **Inset *a*** highlights the absolute optical intensities of the selected CTZ analogues according to pHs. The asterisks highlight the suppressed optical intensities by pH. **(B)** Chemical structures of the CTZ analogues, which were chosen for their characteristic structural differences. As shown in the model, CTZ analogues bearing a hydroxyl (OH) group at the C-2 position (①, ③, ⑱) are heavily suppressed in a high pH range (green circles), whereas a CTZ analogue without an OH group (⑲) at the C-2 position was relatively unaffected by pHs.

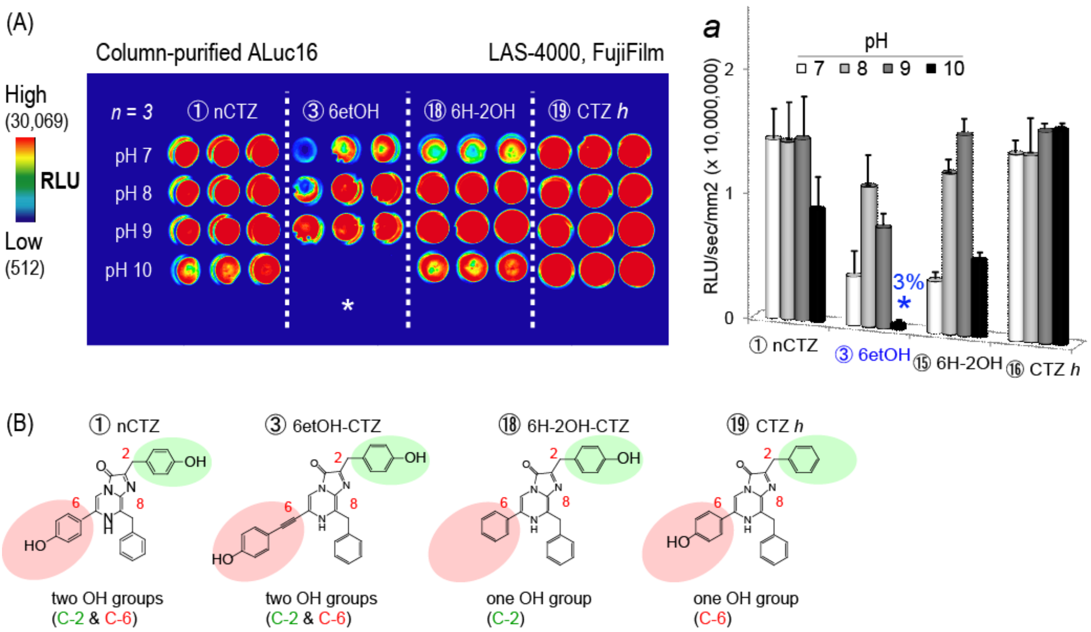

Suppl. Figure 8. Relative autoluminescence of the newly synthesized CTZ analogues. The optical intensities were integrated for 10 seconds with a luminometer (GloMax 20/20n, Promega) immediately after mixing 45 mL of DMSO with 5 mL of each substrate.

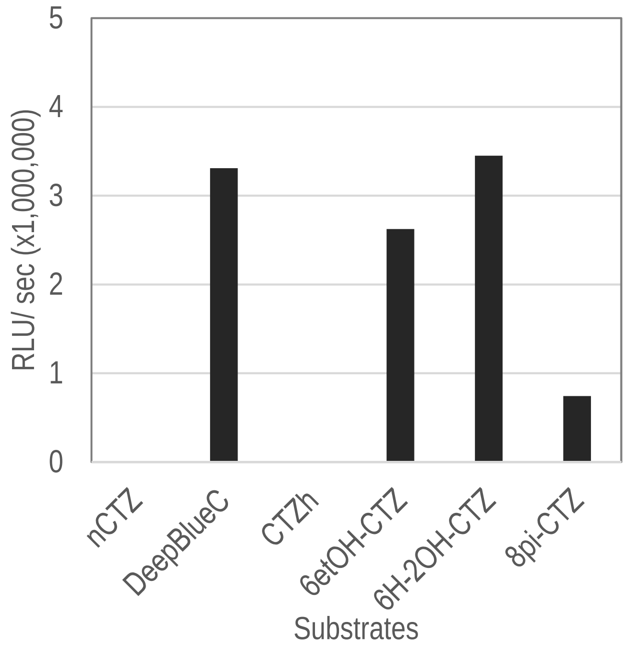

Suppl. Figure 9. Basic scheme for the fabrication of artificial luciferases (ALucs). The sequence was fabricated by extracting frequently occurring amino acids from the alignment of copepod luciferaases in public databases. (A) The prototype sequence of ALucs, which was highlighted in box and red. In case of Region B, we tried to increase the homology between the upper and lower region. This was modified from our previous study [6]. (B) Sequential structures of the artificial luciferases used in the present study: i.e., ALuc16, ALuc23, and ALuc30. The sequence of each ALuc is exactly folded in three. The sequential homology between second and third lines is high.

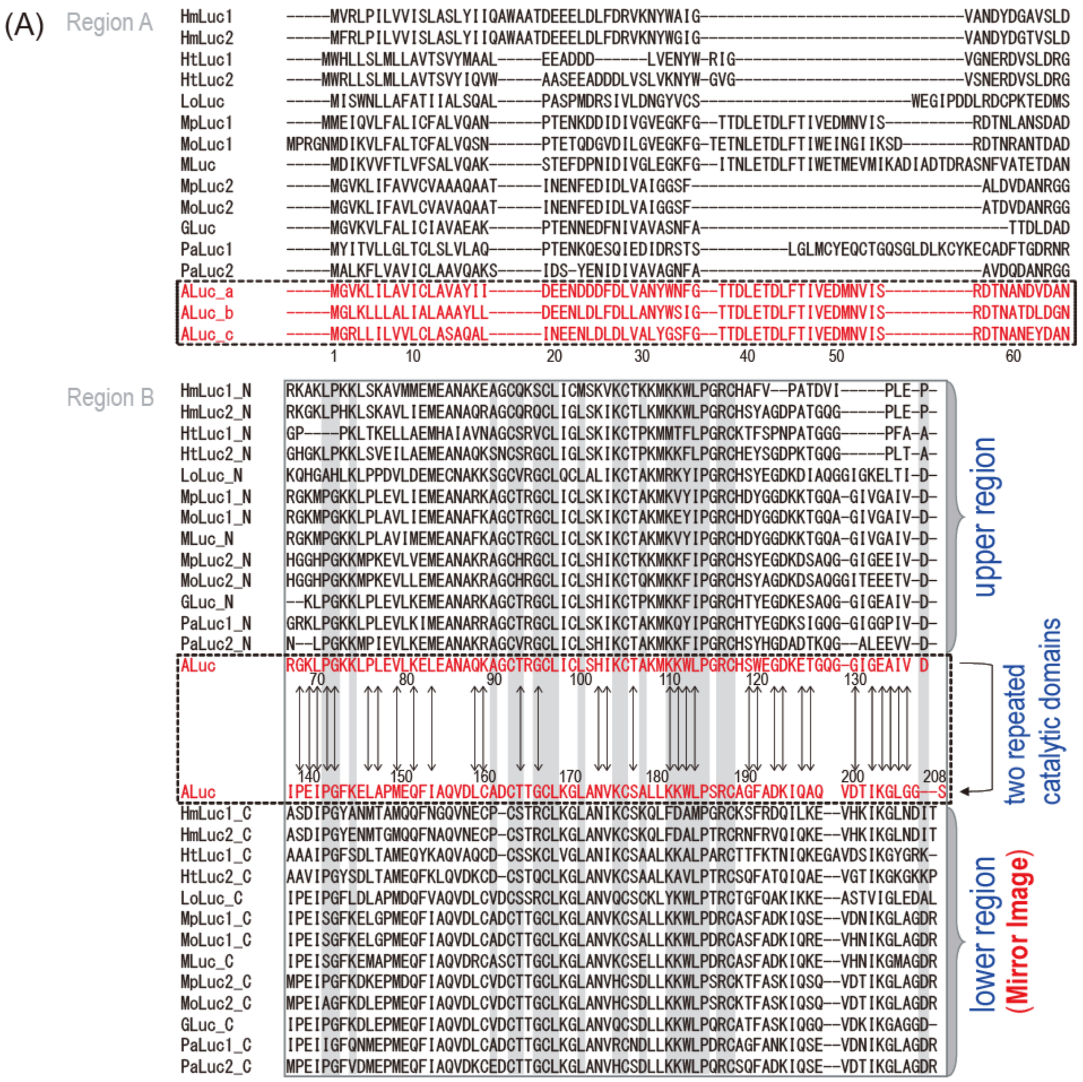

(B)

Sequence of ALuc16

Three stories / Highly conserved

```
-MMGIKVLFALICFALVQANPTENKDDIDIVGVEGKFGTTDLETDLFTIVEDMNVISRDTDVDANRADRGR
RGKLPGKKLPLEVLKELEANAQKAGCTRGCLICLSHIKCTAKMKKWLPGRCESWEGDKETGQGGIGEAIVD
IPEIPGFKDLEPMEQFIAQVDLCVDCTTGCLKGLANVKCSDLLKKWLPSRCATFASKIQAQVDKIKGAGGS
```

Sequence of ALuc23

Three stories / Highly conserved

```
-MMGIKVLFALICFALVQANPTENKDDIDIVGVEGKFGTTDLETDLFTIVEDMNVISRDTDVDANRADRGR
RGKLPGKKLPLEVLKELEANAQKAGCTRGCLICLSHIKCTAKMKKWLPGRCESWEGDKETGQGGIGEAIVD
IPEIPGFKELAPMEQFIAQVDLCADCTTGCLKGLANVKCSALLKKWLPSRCAGFADKIQAQVDTIKGAGGS
```

Sequence of ALuc30

Three stories / Highly conserved

```
-MMGIKVLFALICFALVQANHHHHHHHHDIVGVEGKFGTTDLETDLFTIVEDMNVISRDTDVDANRADRGR
RGKLPGKKLPLEVLKELEANAQKAGCTRGCLICLSHIKCTAKMKKWLPGRCESWEGDKETGQGGIGEAIVD
IPEIPGFKELAPMEQFIAQVDLCADCTTGCLKGLANVKCSALLKKWLPSRCAGFADKIQAQVDTIKGAGGS
```

Supplementary Methods

pH-driven optical specificity of CTZ analogues to marine luciferases. Further, a pH-driven feature of the synthesized CTZ analogues was investigated with a universal buffer. We first determined the optical intensities of luciferases in the full pH range to see the optical profile according to pH (Suppl. Figure 6) and then focused on a narrow range from pH 7 to 10 (Suppl. Figure 7).

For the experiment of Suppl. Figure 6, COS-7 cells grown in the 96-well plate were transiently transfected with pcDNA 3.1(+) vector encoding GLuc, RLuc8.6-535, ALuc23 or ALuc34, and incubated in a CO_2 incubator (Sanyo). Sixteen hours after incubation, the cells were lysed with a lysis buffer (Promega) for 20 minutes according to the manufacturer's instructions. Separately, aqueous universal buffer solutions pH = 4–10 were prepared by mixing the acidic and basic buffer components: i.e., 0.2 M boric acid (H_3BO_3), 0.05 M citric acid, and 0.1 M Na_3PO_4, and the pHs were adjusted with NaOH. 10^{-3} M of nCTZ, 6piOH-CTZ, 6etOH-CTZ, 6H-2OH-CTZ, and CTZ*h* were 10 fold-diluted with the prepared universal buffer solutions with varying pH 4–10 (final concentration: 10^{-4} M), respectively, immediately before the experiments. For the optical measurements, an aliquot of each lysate (10 μL) on a fresh 96-well microplate was simultaneously mixed with 100 μL universal buffer solutions pH 4–10 dissolving 10^{-4} M of nCTZ or 6piOH-CTZ (final concentration) using a multichannel micropipette (Gilson). The microplate was then immediately placed in the dark chamber of the LAS-4000 (FujiFilm), and the corresponding optical intensities were determined and analyzed with Image Reader v2.0 and Multi Gauge v3.1 software (Suppl. Figure 6).

The column-purified ALuc16 in Suppl. Figure 7 was obtained from our previous study [19]. Briefly, a pOPTHM vector encoding ALuc16 (providing a cleavable N-terminal His_6-MBP tag) was expressed in the bacterial strain SHuffle T7 Express *lysS* (New England Biolabs) with 0.3 mM IPTG induction at 16°C. The supernatant of the lysate was purified with an ÄKTA Purifier system (GE Healthcare), and was dialyzed to a metal-cation-free Tris-HCl buffer (0.05 M, pH 8.2) at 4°C for 24 h, and finally adjusted to a concentration of 1 mg/mL by dilution. The purified ALuc16 stock was diluted 500-fold to 2 μg/mL with pure water before the experiments. The measurement of the optical intensities from the

column-purified ALuc16 was conducted as follows. An aliquot of the column-purified ALuc16 (5 µL) was simultaneously mixed with 100 µL universal buffer solutions pH 7–10, dissolving 10^{-4} M of nCTZ, 6etOH-CTZ, 6H2OH-CTZ, or CTZ*h* using a multichannel micropipette (Gilson) on a 96-well optical bottom microplate (Nunc), and the developed optical intensities were determined and analyzed with the image analyzer, LAS-4000 (FujiFilm).

The CTZ analogues exert distinctive luciferase selectivity according to pH ranges. The optical intensities of ALuc23 and ALuc34 in lysates were heavily suppressed to the background intensity in an acidic pH span lower than pH 5 (Suppl. Figure 6), and in the highly basic pH range above pH 11 (data not shown). The maximal optical intensities of nCTZ and 6piOH-CTZ with ALucs were found at pH 8 and 9, respectively.

For determining the precise pH-driven feature of ALucs, ALuc16 was expressed in *E. coli* and was column-purified (Suppl. Figure 7), and the pH-driven optical feature was highlighted in the pH 7–10 range. The optical intensities of ALuc16 with 6etOH-CTZ and 6H-2OH-CTZ were found to be greatly influenced by varying pH, whereas the intensities of nCTZ and CTZ*h* were relatively stable and unaffected by varying pH. Interestingly, the optical intensity of ALuc16 with 6etOH-CTZ was suppressed almost to the background at pH 10 (ca. 3% of the maximal intensity at pH 8): i.e., at pH 10, ALuc16 selectively luminesces with nCTZ, 6H-2OH-CTZ, CTZ*h*, but not with 6etOH-CTZ. The result indicates that the pH can act as a key ingredient for postulating a luciferase-specific assay scheme with minimized optical contamination by combining the pH environment with a specific luciferase–CTZ analogue pair.

Discussion on the pH-driven optical intensities of the CTZ analogures. It is intriguing to interpret the dramatic pH-driven feature in the luciferase–CTZ activities (Suppl. Figure 6). It is noted that CTZ analogues bearing multiple OH groups should be more strongly influenced by pH variations than those bearing single or no OH groups. Furthermore, it should be noted that the OH group on the C-2 of CTZ analogues plays a key role for interacting with ALucs, in contrast to the OH group at the C-6 position (Suppl. Figures 7(A)). Hence, as a rule for the ALuc-CTZ activity, it was determined that CTZ analogues

bearing an OH group at the C-2 position (e.g. nCTZ, 6etOH-CTZ, 6H-2OH-CTZ), were more influenced by pH than other analogues that did not contain an OH group at the C-2 position, for example CTZ*h* as shown in (Suppl. Figures 7(A)).

The optical intensity profile in Suppl. Figure 6 reveals that ALuc-CTZ activity is completely suppressed in the acidic pH range, sharply enhanced in the neutral range (pH 7), and reaches a plateau at a weak basic pH range from pH 8 to 9. In the neutral and basic pH ranges, the OH group of CTZ analogues is considered to be deprotonated, and interacting with the residues in the active site of marine luciferases, as the pKa value of phenols in nCTZ is around 10 [17]. This view is supported by a previous study, where the deprotonated OH group forms a triad bond with the active site residues of luciferase, and acts as an activity center for marine luciferase [13]. Thus, the dramatic pH-driven features of ALuc-CTZ activity may be explained by the pH susceptibility of the OH groups of CTZs deprotonated at each pKa value.

References

1. Adamczyk, M., Johnson, D.D., Mattingly, P.G., Pan, Y. & Reddy, R.E. Synthesis of coelenterazine. *Org Prep Proced Int* **33**, 477-485 (2001).
2. Adamczyk, M. et al. Synthesis of 3,7-dihydroimidazo[1,2a]pyrazine-3-ones and their chemiluminescent properties. *Tetrahedron* **59**, 8129-8142 (2003).
3. Phakhodee, W., Toyoda, M., Chou, C.M., Khunnawutmanotham, N. & Isobe, M. Suzuki-Miyaura coupling for general synthesis of dehydrocoelenterazine applicable for 6-position analogs directing toward bioluminescence studies. *Tetrahedron* **67**, 1150-1157 (2011).
4. Jiang, T.Y. et al. Novel bioluminescent coelenterazine derivatives with imidazopyrazinone C-6 extended substitution for Renilla luciferase. *Org. Biomol. Chem.* **14**, 5272-5281 (2016).
5. Ogawa, T. et al. Synthesis and biological evaluation of p-carborane bisphenols and their derivatives: Structure-activity relationship for estrogenic activity. *Bioorg. Med. Chem.* **17**, 1109-1117 (2009).
6. Kim, S.B., Torimura, M. & Tao, H. Creation of artificial luciferases for bioassays. *Bioconjugate Chem.*, DOI: 10.1021/bc400411h (2013).
